# Surprising Route to a Monoazaporphyrin and Full Characterization of Its Complexes with Five Different 3d Metals

**DOI:** 10.1021/acs.inorgchem.4c00436

**Published:** 2024-04-17

**Authors:** Arik Raslin, Zachary P. Sercel, Natalia Fridman, Irena Saltsman, Zeev Gross

**Affiliations:** † Schulich Faculty of Chemistry, Technion−Israel Institute of Technology, Haifa 32000, Israel; ‡ Division of Chemistry and Chemical Engineering, California Institute of Technology, Pasadena, California 91125, United States

## Abstract

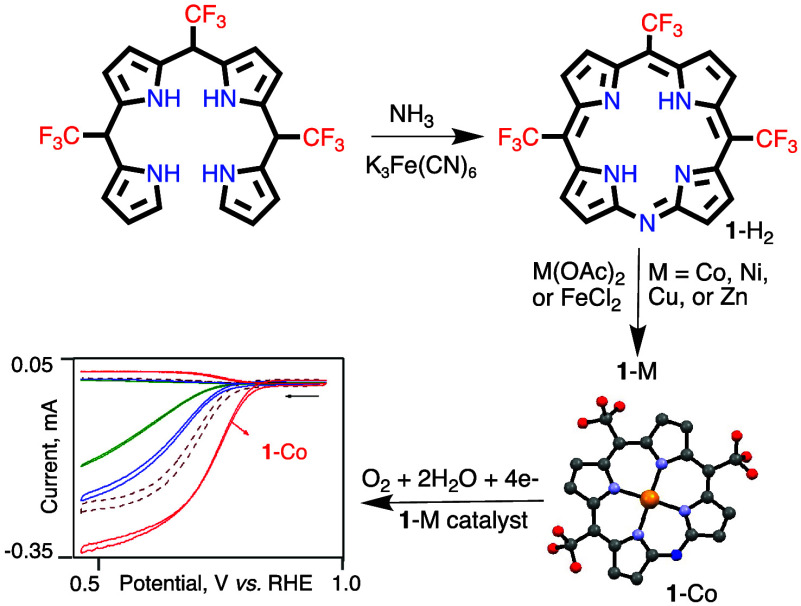

In the search for mild agents for the oxidative cyclization of tetrapyrromethane to the corresponding corrole, we discovered a route that leads to a monoazaporphyrin with three *meso*-CF_3_ groups. Optimization studies that allowed access to appreciable amounts of this new macrocycle paved the way for the preparation of its cobalt, copper, nickel, zinc, and iron complexes. All complexes were fully characterized by various spectroscopic methods and X-ray crystallography. Their photophysical and electrochemical properties were determined and compared to those of analogous porphyrins in order to deduce the effect of the peripheral N atom. Considering the global efforts for designing efficient alternatives to platinum group metal (PGM) catalysts, they were also absorbed onto a porous carbon electrode material and studied as electrocatalysts for the oxygen reduction reaction (ORR). The cobalt complex was found to be operative at a quite positive catalytic onset potential and with good selectivity for the desirable 4-electrons/4-protons pathway.

## Abstract 2

Attempts to improve the synthesis of the tris-CF_3_-substituted corrole led to the discovery of a new monoazaporphyrin, substituted by *meso*-C trifluoromethyl groups and lacking any β-substituents. The effect of the unique N atom on the photophysical and redox properties was determined by comparison of its fully characterized metal complexes with those of porphyrins and corroles with otherwise identical substitutions. The monoazaporphyrin cobalt complexes were deduced to be a good electrocatalyst for the oxygen reduction reaction.

## Introduction

The ever-increasing interest in modified porphyrins may be traced back to the synthesis of the N_4_ macrocycles that serve as prosthetic groups in heme, chlorophylls, Vitamin B_12_, and other natural systems. These tremendous efforts were led by prominent scientists including R. Willstatter, H. Fisher, and R. B. Woodward, whose contributions were recently outlined by Senge et al. in an excellent review.^1^ Research activity on non-natural porphyrins and related macrocycles continues to flourish because of their relevance to fundamental science, including the study of aromaticity and redox noninnocence, as well as for diverse practical applications.^2−4^ A partial list includes drug discovery/development, biomimetic catalysis, and clean energy processes.^5−18^ This also holds true for derivatives in which the metal-coordinating core is modified, being composed of less or more than four N atoms and/or by non-nitrogen elements,^6,10,13^ as well as for those bearing a different macrocyclic skeleton.^5,14,16,19^ Regarding the latter class, the largest advances are arguably in corroles, N_4_ macrocycles with one direct bond between two pyrrole subunits like in the prosthetic group of Vitamin B12 but fully conjugated and aromatic like in classic porphyrins.^19,20^ One major reason for the interest in corroles is that the seemingly small differences relative to porphyrins, at about 10% reduction in the size of the coordination core and the presence of three rather than two protic nitrogen atoms therein, induce very significant changes in the reactivity/stability of the corresponding metal complexes.^3,5,8^ The advantageous outcomes of these features have been outlined in quite a few contemporary reviews, which also include recent synthetic breakthroughs, such as access to minimally substituted corroles.^9,12,21,22^

**Scheme 1:**
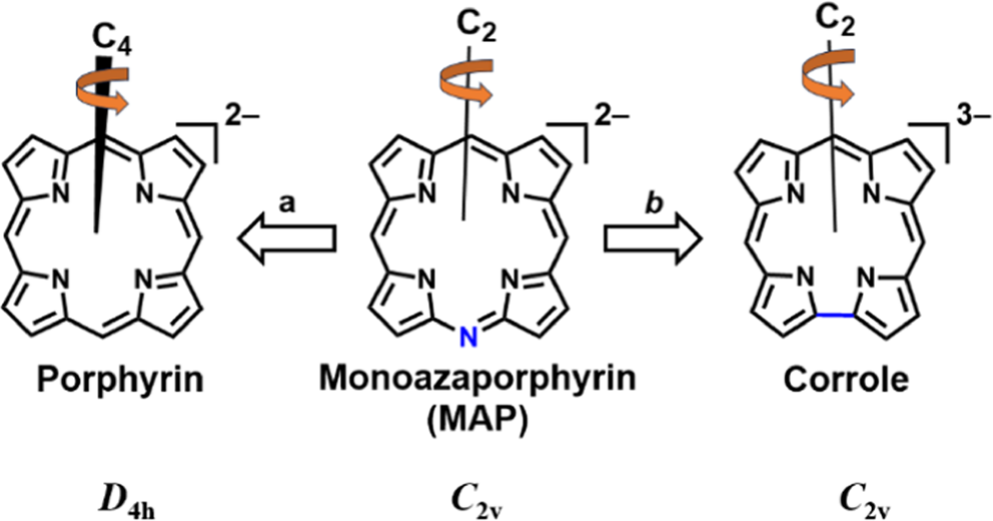
Fully NH-Deprotonated Porphyrin, Monoazaporphyrin (MAP), and Corrole for Emphasizing the Analogy of MAP to (a) the Former in Terms of Charge and (b) the Latter by Virtue of Identical Symmetry

One additional and less emphasized possible difference between corroles and porphyrins is the largest possible symmetry axis in the corresponding metal complexes, *C*_2_ and *C*_4_, respectively. This affects not only spectroscopic features but also allows for selective substitution of the macrocyclic CH protons, as in the case of the 2,17-bis-sulfonated derivative which continues to be the most intensively studied corrole for medicinal applications.^23,24^ An interesting hybrid case is presented by monoazaporphyrins (MAPs), with nitrogen instead of one *meso*-C-R position (R = H, alkyl, or aryl) of porphyrins since they have the coordination core of porphyrins and the symmetry of corroles (Scheme 1). Synthesis of the first MAP was reported as early as 1936,^25^ followed by sporadic rational approaches for improving the synthetic accessibility of these macrocycles.^26−32^ Despite these reports, MAPs have been quite neglected in the field as compared not only to porphyrins, phthalocyanines, and the related tetraazaporphyrins but also relative to corroles. Hitherto reported MAP derivatives are substituted by either alkyls in all eight β-pyrrole positions or (in a singular case only) by aryls on the three *meso*-C atoms, and their coordination compounds formed via chelation of divalent zinc and copper, trivalent iron, and pentavalent iridium and phosphorus.^33−35^ Recent research has focused mainly on spectroscopic features,^33^ but there is one example regarding outstanding reactivity: Neya et al. reported a 50 times higher oxygen affinity of a MAP-reconstituted myoglobin relative to the native protein.^36^

We now report how an incidental finding during attempted improvement of a corrole synthesis led to facile access to a novel MAP derivative with three CF_3_ substituents on the *meso*-C positions. This was followed by the preparation and characterization of zinc(II), nickel(II), copper(II), cobalt(II), and several iron(III) complexes of the macrocycle. The spectroscopic and redox properties of these complexes were compared to those of analogous porphyrins with either H or CF_3_ substituents on C5. Also included is the first investigation of the utility of MAP transition metal complexes as electrocatalysts, which revealed that the cobalt(II) complex is quite a potent and selective catalyst for the oxygen reduction reaction (ORR).

## Results and Discussion

**Scheme 2:**
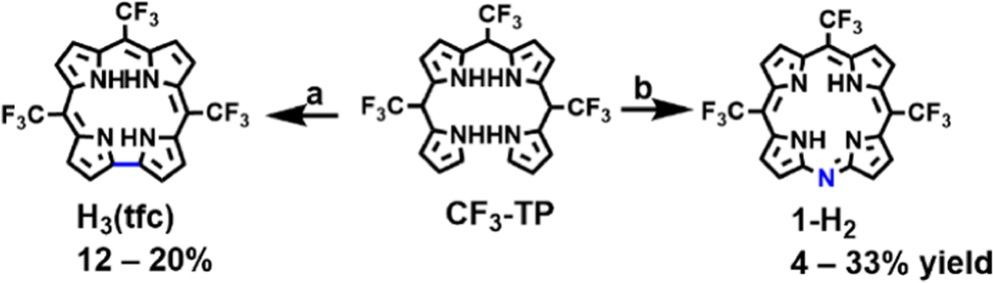
Oxidation of the Tris-CF_3_-Substituted Tetrapyrromethane, **CF**_**3**_**-TP**, by (a) 2 Equivalent of PIFA in Acetonitrile, Leading to the Corrole H_3_(tfc)^21^ and (b) 4 Equivalent of K_3_Fe(CN)_6_ in Aqueous NH_4_OH/NH_3_/CH_3_OH that Led to Monoazaporphyrin **1-H**_**2**_

One conclusion from research that focused on gaining efficient access to the tris-CF_3_-substituted corrole H_3_(tfc) (also for the much more sensitive parent corrole) was that better results are obtained when the oxidant used in the last step is milder than the commonly employed DDQ.^12,23^ Using PIFA, the oxidative cyclization of the CF_3_-substituted tetrapyrrane **CF**_**3**_**-TP** afforded H_3_(tfc) in 12–20% yield (Scheme 2, pathway a), which initiated our search for even milder oxidants. One of the examined options was an aqueous ammonia/methanol solution of K_3_Fe(CN)_6_, which has previously been used to affect the oxidative cyclization of a biladiene to a corrole.^37^ Much to our initial surprise, the sole isolable product was not the expected corrole but monazaporphyrin **1-H**_**2**_ (Scheme 2, pathway b). A search of the corrole literature revealed that the first-ever corrole synthesis also reported a (not fully characterized) MAP as a minor byproduct.^38^ The new **1-H**_**2**_ is novel by means of having C1 substituents on the *meso*-C atoms and unsubstituted β-pyrrole positions. There is only one similar MAP derivative, the iridium complex with C_6_F_5_ groups on the *meso*-C positions, which was reported by Palmer et al. but not further studied.^39^

The initially employed reaction conditions provided **1-H**_**2**_ in 4% yield and no other isolable macrocycle (entry 1, Table 1). Trying to gear the reaction toward H_3_(tfc), 300 mL of aq. NaOH or K_2_CO_3_ were employed as alternatives for NH_4_OH as bases (entries 2 and 3, Table 1), but neither corrole nor porphyrin was formed in detectable amounts. Reducing the volume of methanol, required for dissolving precursor **CF**_**3**_**-TP**, from 50 to 15 mL and thus increasing the amount of dissolved ammonia, raised the yield of the MAP from 4 to 8% (Table 1, entry 4). A further jump in the yield of **1-H**_**2**_ to 15% was achieved by replacing regular methanol with a 7N NH_3_ methanol solution (Table 1, entry 5). Experiments in NH_3_/MeOH only, with no aqueous ammonia, did not yield any detectable product. This clearly shows that NH_4_OH is a critical cosolvent for dissolving the inorganic oxidant K_3_Fe(CN)_6_, but it is also oxidized and provides nitrogen for MAP formation. The best results were obtained by reducing the temperature of the reaction to 60 °C (Table 1, entry 4), yielding **1-H**_**2**_ in a respectable 33% yield. Considering the price and long-term stability of 7N NH_3_/CH_3_OH solutions, an alternative ammonia precursor was sought for. The choice was ammonium carbamate, the solid and cheap material that is very soluble in methanol and was previously reported for different nitrogen insertion processes.^40^ With 26 equiv of ammonium carbamate as an additive, **1-H**_**2**_ was obtained in 26% chemical yield (Table 1, entry 7)

**Table 1:** Yields of **1-H**_**2**_ Obtained by Variation of the Reaction Conditions^a^

entry	cosolvent	temperature	% yield
1	50 mL of CH_3_OH	reflux	4
2^b^	50 mL of CH_3_OH	reflux	0^d^
3^c^	50 mL of CH_3_OH	reflux	0^d^
4	15 mL of CH_3_OH	reflux	8
5	15 mL of 7N NH_3_/CH_3_OH	reflux	15
6	15 mL of 7N NH_3_/CH_3_OH	60 °C	33
7	15 mL of CH_3_OH + 760 mg of ammonium carbamate	60 °C	26

a Common to all reactions, except of entries 2 and 3, is that they were performed by using potassium hexacyanoferrate (4 equiv) in aqueous ammonia solution (300 mL) as an oxidant.

b Aqueous NaOH is used instead of aqueous ammonia.

c Aqueous K_2_CO_3_ is used instead of aqueous ammonia.

d Neither **1-H**_**2**_ nor H_3_(tfc) is formed.

Free-base **1-H**_**2**_ and the corresponding metal complexes (*vide infra*) were fully characterized by the combination of high-resolution mass spectrometry (HRMS) (Figures S1–S7) and NMR spectroscopy (Figures S8–S19). The corrole-like *C*_2_ symmetry axis leads to the occurrence of four resonances (doublets) corresponding to the eight β-pyrrolic CH protons and two ^19^F resonances in a 2:1 ratio for the two kinds of CF_3_ groups in the ^1^H and ^19^F NMR spectra, respectively (Figures S8–S9). The NH protons of **1-H**_**2**_ appear at a chemical shift of −2.5 ppm and are as sharp as in regular porphyrins and not very broad as in corroles (Figure S8).^41^

**Figure 1:**
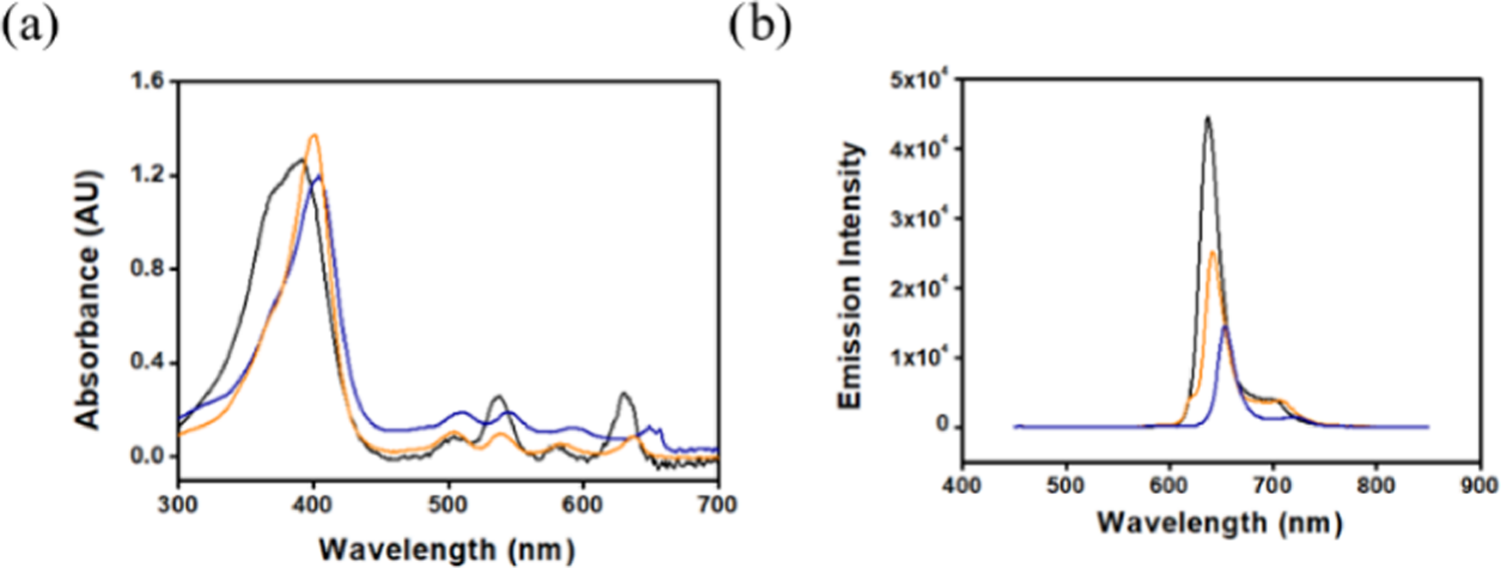
Comparison of the (a) electronic spectra and (b) emission spectra (optically matched, *i.e.*, identical absorbance at the excitation wavelength of 400 nm) of **1-H**_**2**_ (black), **2-H**_**2**_ (orange), and **3-H**_**2**_ (dark blue) in CH_2_Cl_2_.

**Scheme 3:**
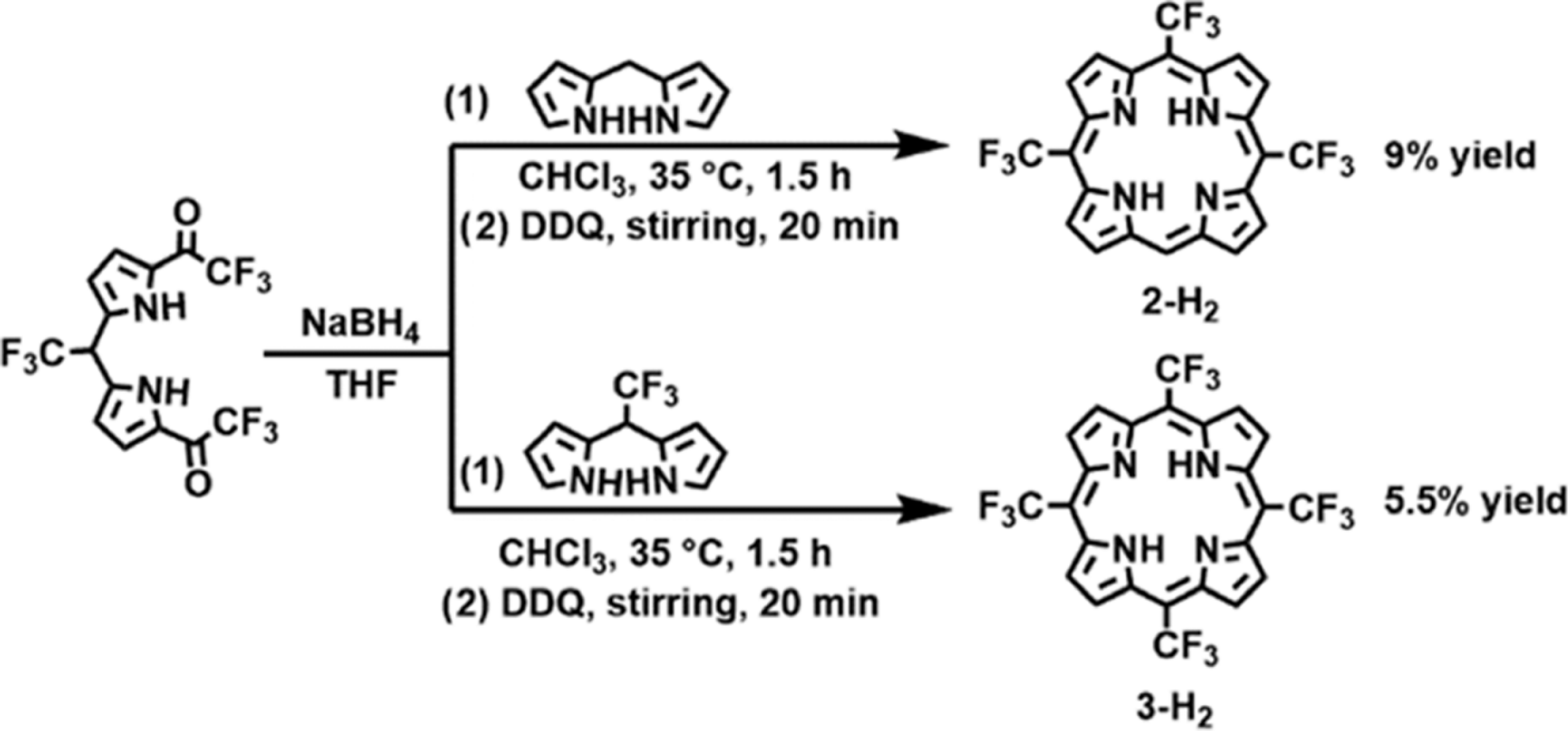
Synthesis of Porphyrins **2-H**_2_ and **3-H**_**2**_ as Analogs of New MAP **1-H**_**2**_

Insights into the effect of the *meso*-N atom on the photophysical and redox properties of the new MAP required the synthesis of related porphyrins, with either CH or CCF_3_ at the position occupied by a nitrogen atom in **1-H**_**2**_. Compounds **2-H**_**2**_ and **3-H**_**2**_, which have the same symmetry as **1-H**_**2**_ and regular porphyrins, respectively, were prepared by slight modifications of previously reported procedures (Scheme 3).^42,43^ The electronic spectra revealed typical near-UV (Soret) and four visible (Q) bands for all compounds (Figure 1a). But **1-H**_**2**_ differs from the two regular porphyrins by (a) a significantly broader Soret band and (b) by alternating intensity of the Q bands rather than a gradual decrease in their absorbance.^43^ The substitution of the methine bridge present in **2-H**_**2**_ and **3-H**_**2**_ by the N atom in **1-H**_**2**_ induces a significant increase in the intensity of two of the four Q bands in the electronic spectra, similar to the phylloporphyrin, for which this phenomenon was originally discovered and found to be highly dependent on the *meso-C* substituents.^44−46^ The 400 nm excitation of optically matched dichloromethane solutions revealed that the emission intensity of **1-H**_**2**_ is 1.5 and 3.3 times larger than those of **2-H**_**2**_ and **3-H**_**3**_, respectively (Figure 1b). This phenomenon likely reflects greater rigidity and fewer degrees of freedom when the position that differs among these three macrocycles bears a nitrogen atom rather than a CH or CCF_3_ unit. The combination of more pronounced Q bands and stronger emission of **1-H**_**2**_ suggests potential photocatalytic applications for this compound and the corresponding metal complexes.

**Scheme 4:**
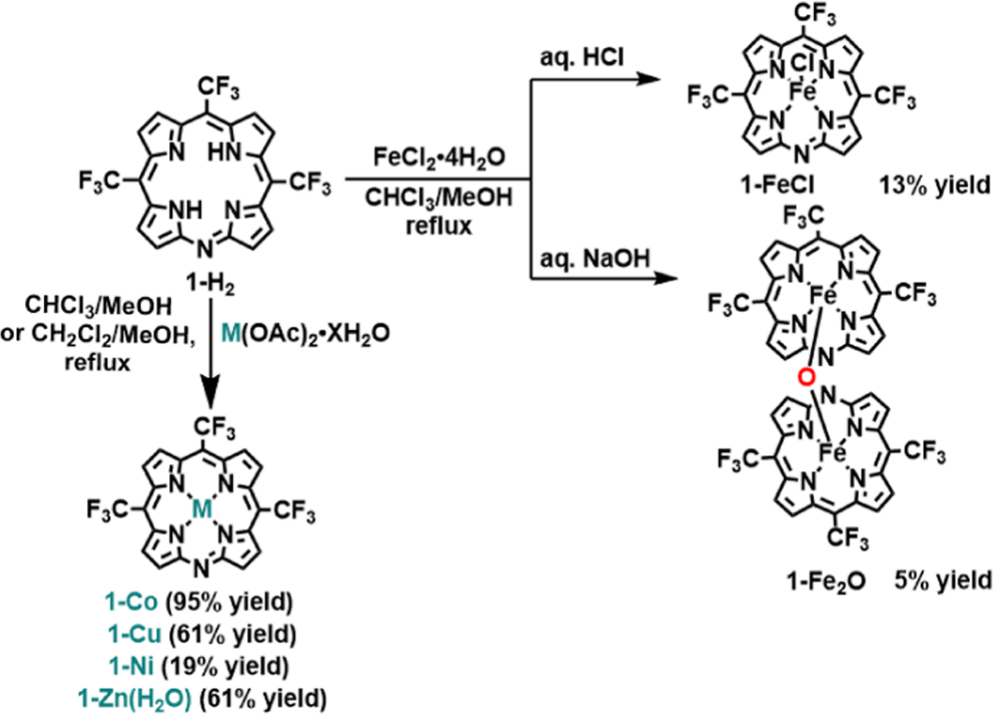
Metalation of **1-H**_**2**_ by Cobalt, Nickel, Copper, Zinc, and Iron

Metalation of **1-H**_**2**_ by the bis-acetates of cobalt(II), nickel(II), zinc(II), and copper(II) under mild reaction conditions allowed for the isolation of **1-Co**, **1-Ni**, **1-Zn(OH**_**2**_**)**, and **1-Cu**, respectively (Scheme 4, bottom). The insertion of iron (with FeCl_2_ as a metal source) provided a mixture of mono- and binuclear complexes, which upon washing the organic solution with either aq. HCl or aq. NaOH afforded chloro-coordinated **1-FeCl** and μ-oxo-bridged **1-Fe**_**2**_**O**, respectively (Scheme 4, right).

**Figure 2:**
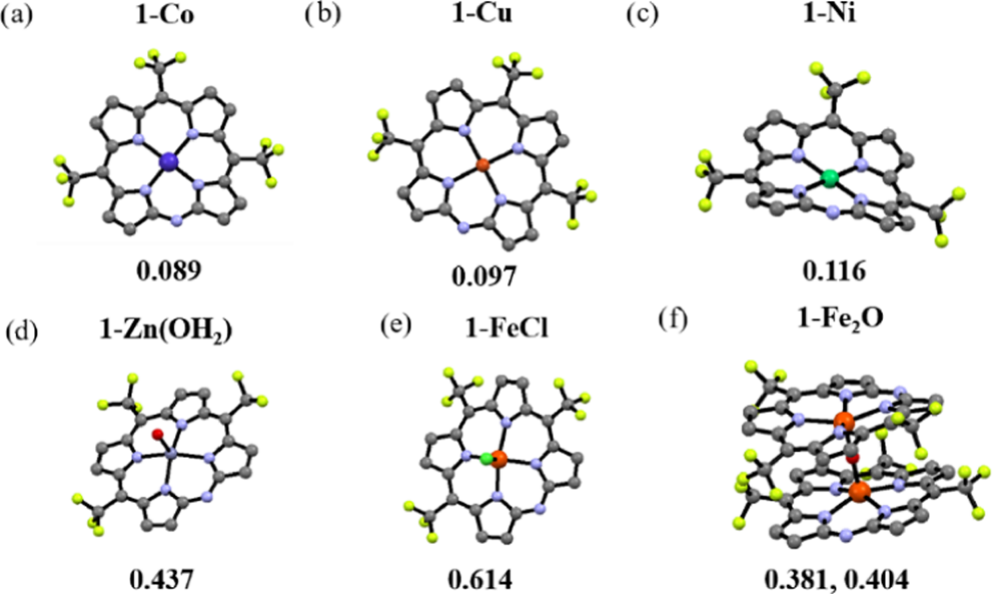
H-omitted presentation of the X-ray structures of the different complexes. Color legend: red—oxygen, yellow—fluorine, blue—nitrogen, green—chlorine, gray—carbon, purple—cobalt, orange—iron, light blue—nickel, black—copper, and light green—zinc. The numbers below the crystals indicate the deviation of the metal center from the 24 atom plane of the macrocycle (Δ*M*^24^) in Å.

X-ray quality crystals were obtained for all of the new MAP complexes as well as for the analogous porphyrin **2-H**_**2**_ (Figure 2). One interesting observation is that not only the d^8^ nickel(II) complex but also the d^7^ cobalt(II) and the d^9^ copper(II) complexes are 4-coordinate. The average metal–N bond lengths are 1.9284, 1.9102, and 1.9650 Å for **1-Co**, **1-Ni**, and **1-Cu**, and the metal ion deviations from the macrocycle plane are 0.089, 0.116, and 0.097 Å, respectively. Comparison with octaethylporphyrinato metal complexes reveals an identical trend in the M–N bond lengths. This has been explained by the difference in metal ion size, which is 0.58, 0.55, and 0.57 Å for Co^II^, Ni^II^, and Cu^II^, respectively, affecting interporphyrin π–π interactions and deviation from planarity.^47,48^ It can be deduced that the MAP cobalt and nickel complexes follow the same trend as their ionic radii, with Co^II^ bigger than Ni^II^, while the difference between the cobalt and copper complexes is due to the larger deviation from the planarity of the latter. The other three complexes are 5-coordinate, with the metal above the N_4_ plane by 0.437, 0.614, and 0.381–0.404 Å for **1-Zn(OH**_**2**_**)**, **1-FeCl**, and **1-Fe**_**2**_**O**, respectively. The metal–N bond lengths are longer than in the four-coordinate complexes, ranging from 2.046 in **1-FeCl** to 2.059 in **1-Fe**_**2**_**O** and 2.063 Å in **1-Zn(OH**_**2**_**)**. The Fe–O–Fe angle in the dimer is 178.13°, almost completely straight. A feature common to all complexes is that the macrocycle is not planar. They adopt the ruffled geometry, with *meso*-C atoms alternately located below and above the macrocycle plane, while all of the pyrroles are tilted to the same direction.^15,49^

**Figure 3:**
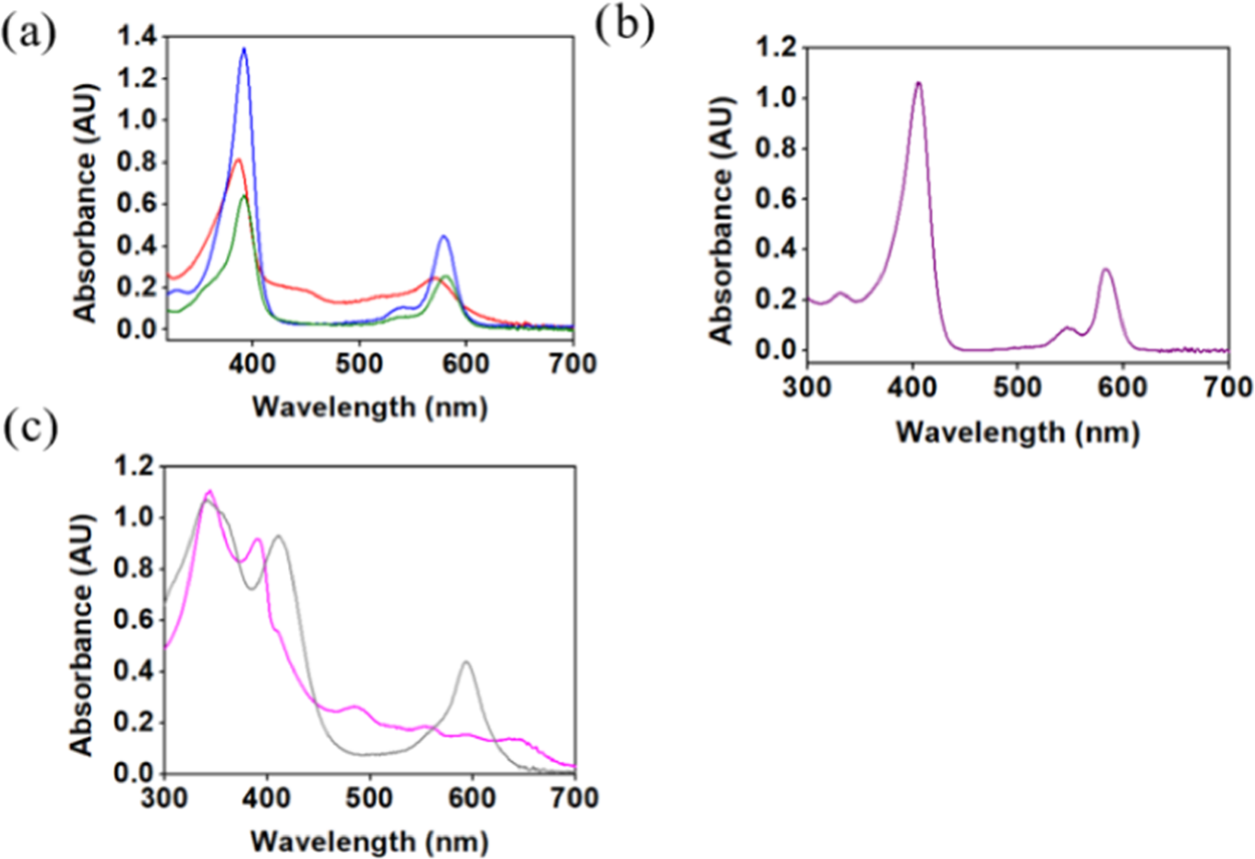
Electronic spectra (in CH_2_Cl_2_) of (a) **1-Co** (red), **1-Cu** (blue), **1-Ni** (green), (b) **1-FeCl** (pink), **1-Fe**_**2**_**O** (gray), and (c) **1-Zn** (purple, in THF).

The electronic spectra of the complexes (Figure 3) were recorded in CH_2_Cl_2_, except for **1-Zn** which was insufficiently soluble and was hence examined in THF. The divalent cobalt, copper, nickel, and zinc complexes displayed “normal” spectra:^50^ a single near-UV (Soret) band with maxima at 389 ± 3 nm and one dominant visible (Q) band at 576 ± 5 nm with a shoulder at about 40 nm shorter wavelengths. (Figure 3a–b). The spectra of the trivalent iron complexes are quite different: mononuclear **1-FeCl** has a split Soret band, a charge transfer band at 488 nm, and three Q bands, while the Soret band of binuclear **1-Fe**_**2**_**O** is even more split and it has only one Q band and no charge transfer band (Figure 3c). HRMS and extensive NMR data are provided in the Supporting Information (SI) (S1–S7 and S8–S18).

**Figure 4:**
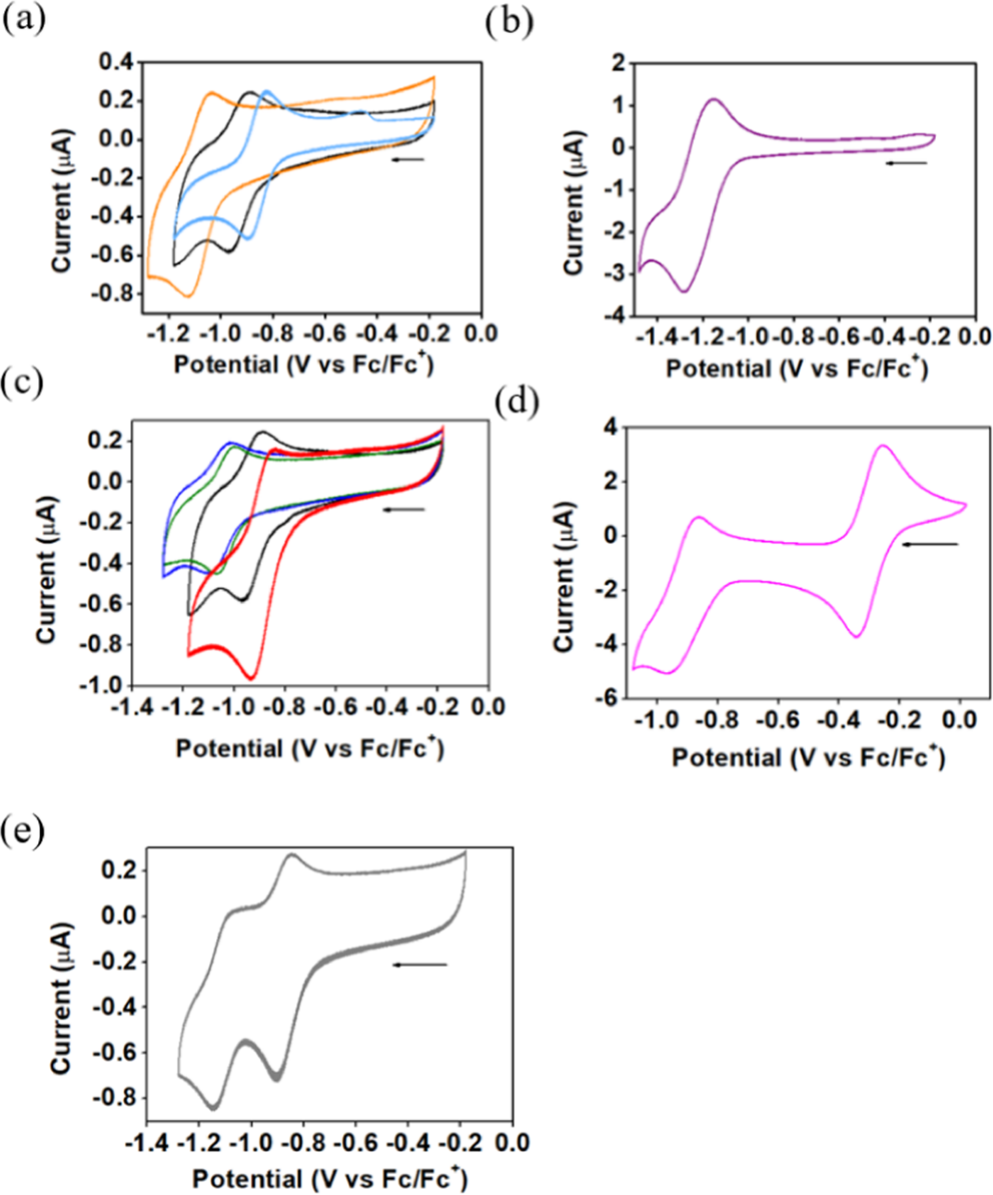
CV traces (0.1 M TBAPF_6_ electrolyte, scan rate = 100 mV/s; N_2_ atmosphere, electrodes: working, glassy carbon; reference, Ag/AgNO_3_; counter, Pt wire) of acetonitrile (CH_2_Cl_2_ for the iron complexes) solutions containing 0.5 mM of (a) **1-H**_**2**_ (black), **2-H**_**2**_ (orange), **3-H**_**2**_ (light blue), (b) **1-Zn(OH**_**2**_**)**, (c) **1-H**_**2**_ (black), **1-Co** (red), **1-Cu** (blue), **1-Ni** (green), (d) **1-FeCl** (pink), and (e) **1-Fe**_**2**_**O** (gray).

Investigation of the redox properties of the new MAP started with a comparison to the analogous porphyrins, which share with **1-H**_**2**_ the presence of three *meso*-C–CF_3_ but have either CH (**2-H**_**2**_) or another C–CF_3_ (**3-H**_**2**_) group rather than an N atom. Circular voltammograms of these metal-free compounds exposed one quasi-reversible process for each (Figure 4a) that corresponds to the reduction and reoxidation of the corresponding macrocycle. The half-wave potentials (*E*_1/2_) were deduced to be −1.07, −0.92, and −0.86 V for **2-H**_**2**_, **1-H**_**2**_, and **3-H**_**2**_, respectively. Considering the differences in the redox potentials relative to **2-H**_**2**_ uncovers that the N atom is more electron-withdrawing than CH (Δ*E*_1/2_ = 0.15 V) and only slightly less than C–CF_3_ (Δ*E*_1/2_ = 0.21 V). These studies were followed by investigating **1-Zn(OH**_**2**_**)**, whose reversible redox event at *E*_1/2_ = −1.21 V (Figure 4b) is safely attributed to ring reduction.

The complexes with potentially redox-active metal ions displayed reversible/semireversible reduction waves with *E*_1/2_ values of −0.88 V for **1-Co** and −1.03 V for both **1-Cu** and **1-Ni** (Figure 4c). This trend is consistent with the reduction of all complexes being macrocycle-centered but influenced by the electronegativity of the chelated metal ion. This variable was deduced by Kadish et al. in the study of many tetraphenylporphyrin metal complexes to be in the order of Zn^2+^ > Cu^2+^ ∼ Ni^2+^ > Co^2+^.^51,52^ Mononuclear iron(III) complex **1-FeCl** displays two reversible redox events (Figure 4d) of which the first, with *E*_1/2_ = −0.29 V, may safely be attributed to an Fe^II^/Fe^III^ couple because it is so much less negative than that of free-base **1-H**_**2**_ and zinc complex **1-Zn**.^53,54^ The second reduction (*E*_1/2_ = −0.91 V) is already at potentials where macrocycle reduction must be considered possible. The case of binuclear oxo-bridged iron(III) complex **1-Fe**_**2**_**O** is more complex since the first and second events appear at *E*_1/2_ = −0.88 V and −1.1 V (Figure 4e), at which both the macrocycle and the metal ion could be redox active. All of the complexes exhibited only irreversible transformations at more negative potentials, which is common for porphyrins not substituted by large aryl groups that protect against decomposition.^34,42^

**Figure 5:**
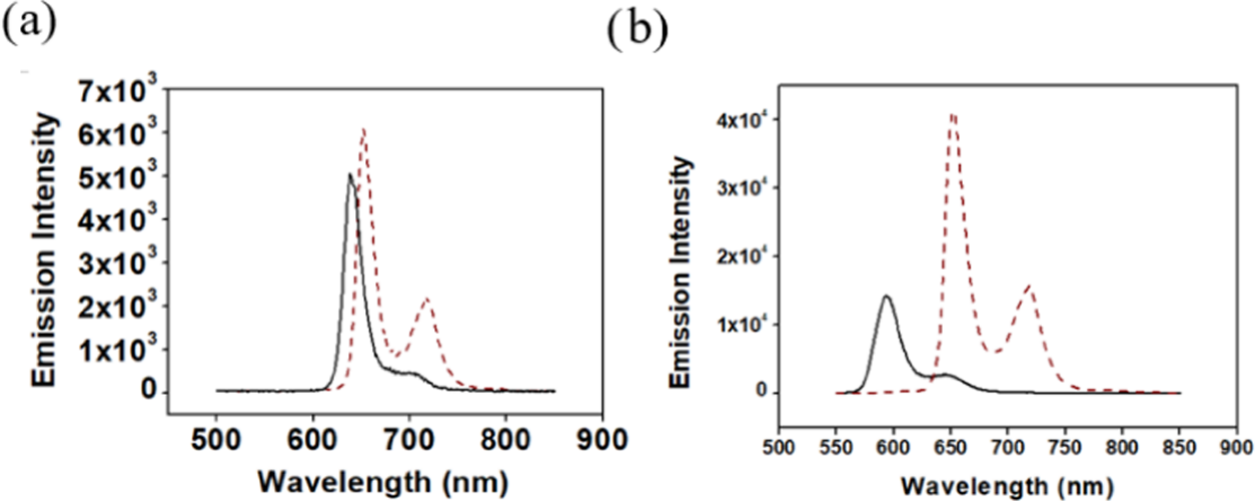
Emission spectra (toluene/aerobic/293 K) of (a) **1-H**_**2**_ (black trace, λ_exc_ = 413 nm), with emission maxima at 640 and 706 nm, and (b) **1-Zn(OH**_**2**_**)** (black trace, λ_exc_ = 415 nm), with emission maxima at 593 and 648 nm, in comparison with that of 5,10,15,20-(tetraphenyl)porphyrin (broken line, λ_exc_ = 415 nm).

Having deduced that the emission intensity of the new MAP is larger than that of porphyrins with identical substituents, the fluorescence quantum yields of **1-H**_**2**_ and **1-Zn(OH**_**2**_**)** were determined. This was done relative to free-base 5,10,15,20-tetraphenylporphyrin (H_2_TPP) (Figure 5), whose quantum yield is 0.11 in toluene under aerobic conditions.^55^ The quantum yields of **1-H**_**2**_ and **1-Zn(OH**_**2**_**)** were deduced to be 0.075 and 0.036, respectively. The latter value is very similar to the quantum yield of ZnTPP (0.033),^55,56^ which is commonly used for photocatalysis and photoredox processes.^55^

**Figure 6:**
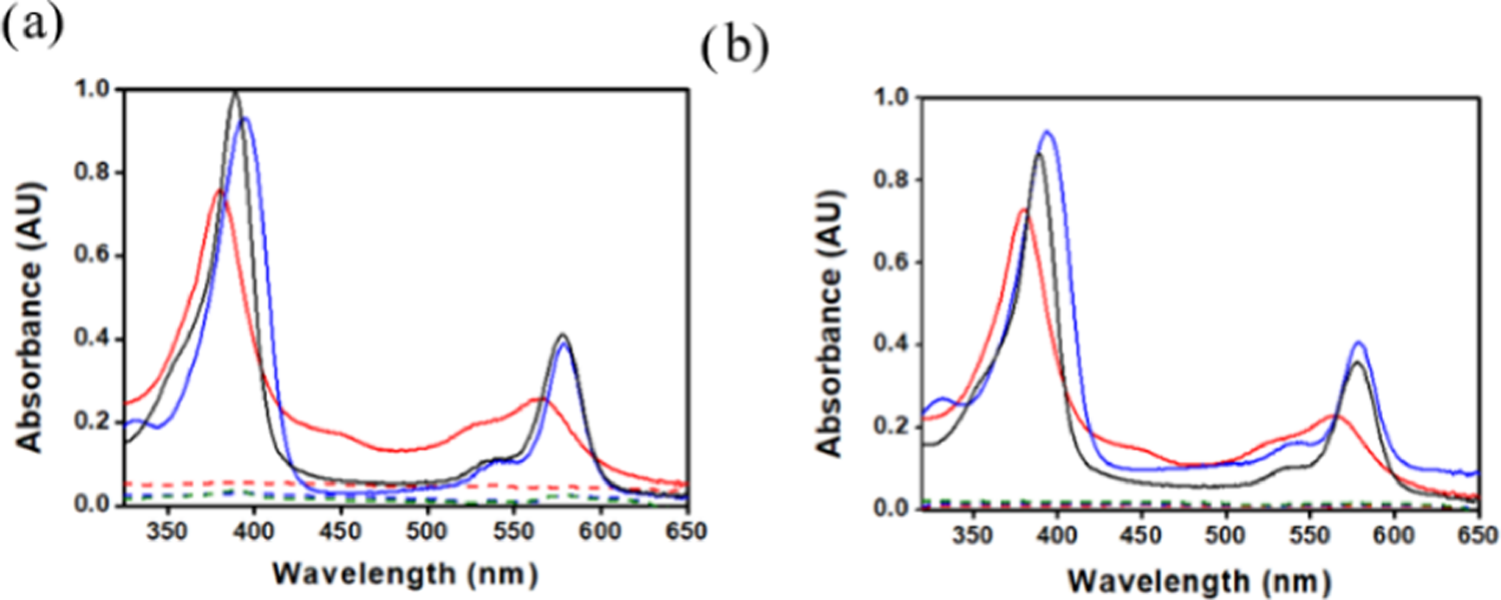
Electronic spectra of 0.8 mg **1-Co** (red), **1-Cu** (blue), and **1-Ni** (black) before (solid lines) and after (dashed lines) adsorption onto 10 mg of (a) Vulcan and (b) BP2000 from 1 mL of isopropanol solutions.

The transition metal complexes of the new MAP were briefly examined for exploring their potential utility as electrocatalysts for the heterogeneous oxygen reduction reaction (ORR), frequently considered as most crucial for the future of fuel cells.^57−59^ A common procedure for such testing is to adsorb the potential catalysts onto porous carbon materials that are conductive, robust, have a high surface area, and guarantee efficient mass and charge transport.^60−64^ Vulcan XC-72R (Vulcan) and Black Pearls 2000 (BP2000) were employed here, noting that the latter has a much bigger surface area (1537 versus 235 m^2^/g) and ten times more mesopores.^65^ The process started by dissolving the molecular catalyst in 2-propanol and mixing it with the insoluble carbon. Both iron complexes were excluded from this study because **1-FeCl** was not stable in isopropanol and **1-Fe**_**2**_**O** was not soluble in it. In contrast with *meso*-aryl substituted porphyrins and corroles, whose adsorption onto BP2000 is much more significant than onto Vulcan,^57^**1-Co**, **1-Ni**, and **1-Cu** were fully adsorbed on both carbons (Figure 6) when mixed in a 0.8/10 mg ratio. This phenomenon may be confidently attributed to the small size of *meso*-CF_3_ relative to *meso*-aryls: the latter are perpendicular to the macrocycle and hence interfere with its π-stacking interactions, which are responsible for the spontaneous physisorption onto the carbon surface.

**Figure 7:**
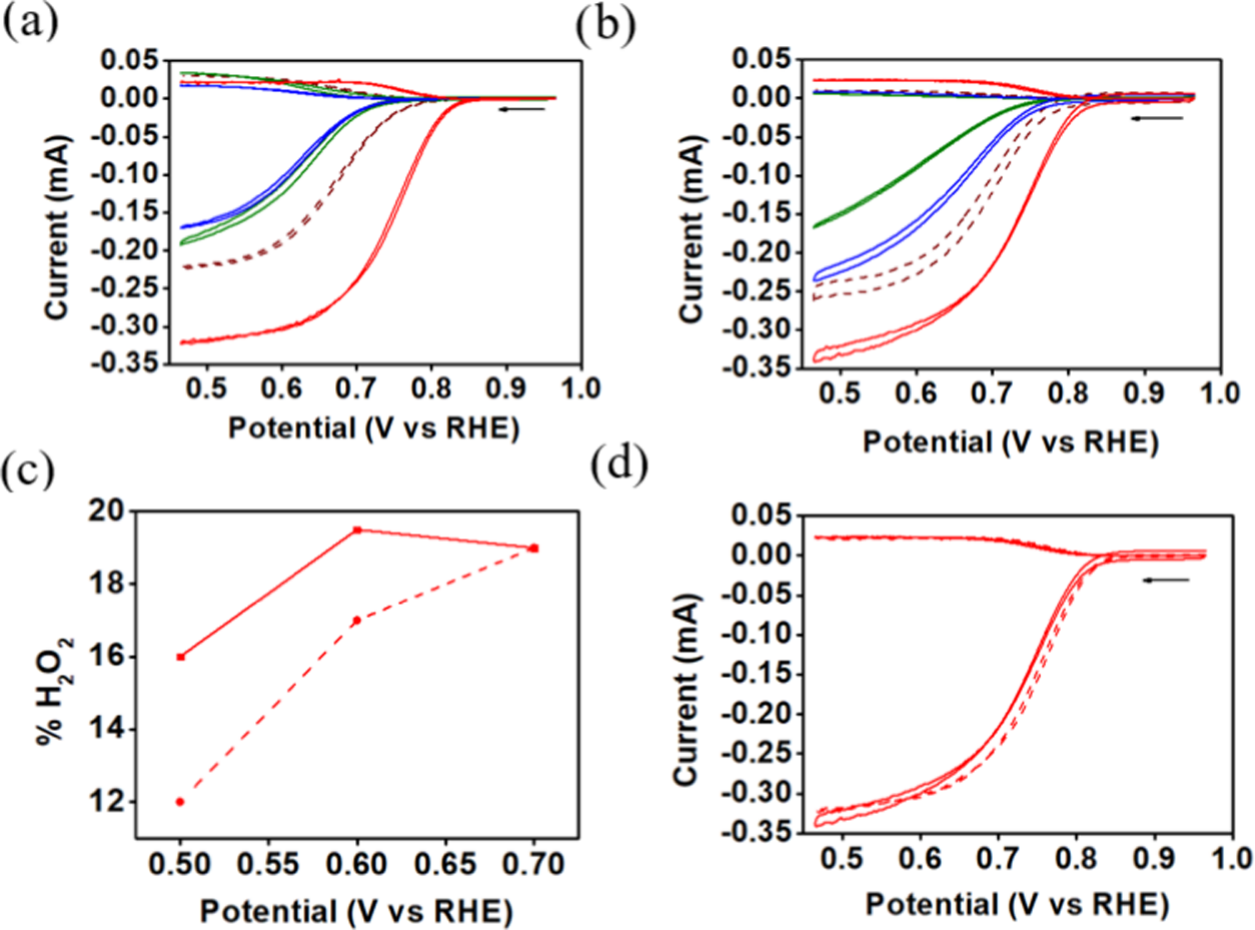
RRDE measurements (0.1 M KOH, Ag/AgCl reference and Pt wire counter electrodes) of **1-Cu** (blue), **1-Ni** (green), and **1-Co** (red) absorbed onto (a) Vulcan and (b) BP2000 (the dashed traces are the nonmodified carbon support); (c) percentage of hydrogen peroxide formed via catalysis by **1-Co**; and (d) comparison of ORR by **1-Co** absorbed onto BP2000 (solid) or Vulcan (dashed).

Studying the azaporphyrin complexes for ORR at pH 13 exposed the nickel and copper complexes as being irrelevant for catalyzing this reaction. They even harmed the performance of the nonmodified carbon electrode, presumably by blocking active sites. **1-Co** was however active when absorbed on either Vulcan or BP2000 (Figure 7a,b), with an onset potential of 0.84 V on both carbons, a half-wave potential (*E*_1/2_) of 0.72 V on BP2000 and 0.74 V on Vulcan, and producing only 12–16% H_2_O_2_ at 0.5 V and 19% at 0.7 V (Figure 7c, percentages calculated by eqs S1). This clearly shows that the metal plays a significant role in this catalysis, as all other parameters (macrocycle size, *meso*-substituents, lack of axial ligands, and adsorption on carbon) are identical and that even the carbon type does not play a significant role (Figure 7d).

**Figure 8:**
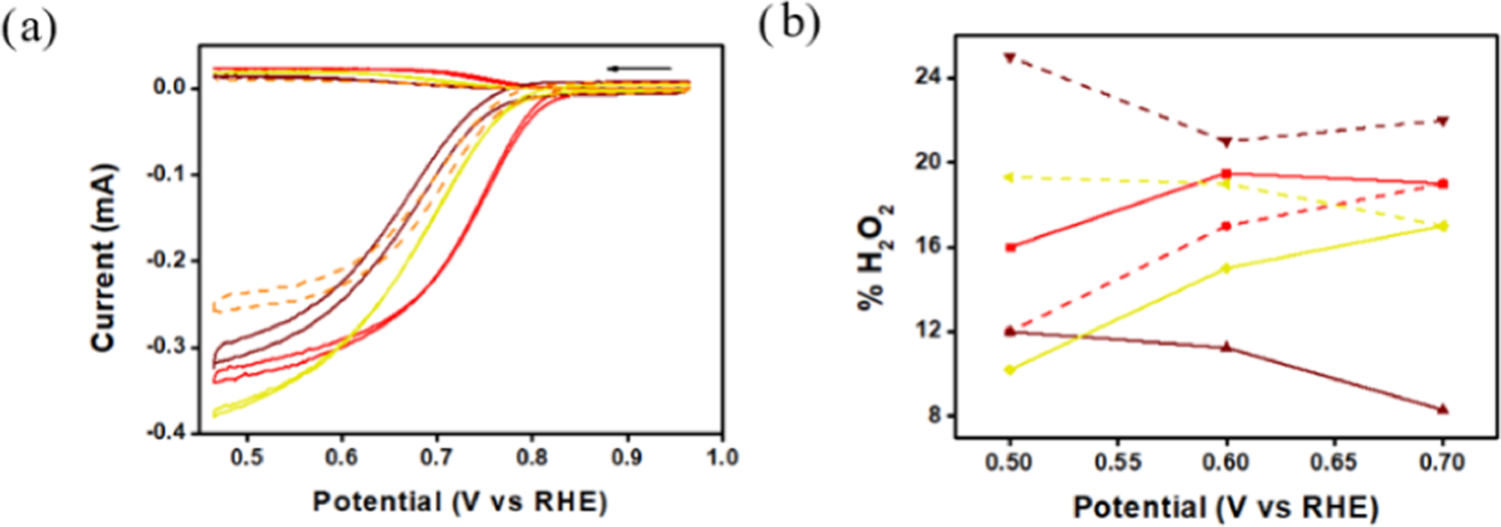
RRDE measurements of (a) **1-Co**@BP2000 (red), **2-Co**@BP2000 (brown), **3-Co**@BP2000 (yellow) in a oxygen-saturated 0.1 M KOH solution, at 1000 rpm, and a scan rate of 20 mV/s, and (b) percentage of H_2_O_2_ formed via catalysis by **1-Co**, **2-Co**, and **3-Co** when adsorbed onto BP2000 (solid) and Vulcan (dashed).

The results obtained with electrode modifications by **1-Co** were briefly compared to those with the cobalt complexes of analogous porphyrins **2**-H_2_ and **3**-H_2_. Both porphyrin complexes (**2-Co** and **3-Co**) were fully adsorbed when treated with either Vulcan or BP2000 (Figure S19), similar to the observation with **1-Co**. Despite the small differences— occupation of the C20 position by C–H, CCF_3_, and N, respectively—the catalytic activity of electrodes modified by them varied significantly (Figure 8). This was revealed by deducing that the superiority of **1-Co**@BP2000 is its onset potential: more positive by 50 and 40 mV relative to **2-Co**@BP2000 and **3-Co**@BP2000, respectively (Figure 8a).

On the other hand, significantly less undesired H_2_O_2_ is formed with the two latter when adsorbed onto BP2000 (Figure 8b). By this criterion, the best results were obtained for catalysis by **3-Co**@BP2000, producing only 10 to 20% H_2_O_2_. Full characterization and further studies of the new cobalt complex, including other electrocatalytic processes of clean energy relevance, will be reported in the near future.

## Conclusions

Introduced is the facile synthesis of a novel free-base monoazaporphyrin with no substituents on the β-pyrrolic atoms and electron-withdrawing trifluoromethyl groups on the *meso*-C positions. The divalent zinc, cobalt, copper, and nickel complexes and both mononuclear and binuclear iron(III) complexes were fully characterized in terms of structures and photophysical and redox properties. Carbon electrodes modified by the cobalt complex were found to be worthy electrocatalysts for heterogeneous oxygen reduction with a quite positive catalytic onset potential and good selectivity for the desirable reduction to water rather than to hydrogen peroxide.

## Experimental Section

### Synthesis of 10,15,20-Tris(trifluoromethyl)-5-azaporphyrin (**1-H**_**2**_**)**

The initial experiments were performed by dissolving 1.4 g (2.8 mmol) of 5,10,15-tris(trifluoromethyl)tetrapyrromethane (**CF**_**3**_**-TP**)^21^ in 50 mL of methanol, followed by adding (3.7 g, 0.01 mol) potassium hexacyanoferrate(III) and 300 mL of 25% NH_4_OH, and heating the solution to reflux for 1.5 h. After it was cooled to room temperature, the reaction mixture was extracted with CH_2_Cl_2_, dried over sodium sulfate, and purified on a silica column with 10% ethyl acetate/hexane as the eluent. The amount of **1-H**_**2**_ was 40 mg in 4% chemical yield.

All of the other experiments were performed with the same amounts of **CF**_**3**_**-TP**, K_3_[Fe(CN)_6_] of aqueous solution but with several differences, attempted to increase the yield of the product:

(a) Replacing ammonium hydroxide by either aqueous K_2_CO_3_ or NaOH did not lead to any corrole or porphyrin.
(b) Decreasing the volume of methanol from 50 to 15 mL, with addition of K_3_[Fe(CN)_6_] and 300 mL of NH_4_OH and heating to reflux, lead to 8% yield.
(c) Using 15 mL of 7 N NH_3_/CH_3_OH as an organic solvent for **CF**_**3**_**-TP** instead of methanol and 2 h reflux increased the yield to 15%.
(d) The best conditions were achieved when **CF**_**3**_**-TP** (160 mg, 0.315 mmol) was dissolved in 15 mL of 7 N NH_3_/methanol. Potassium hexacyanoferrate(III) (415 mg, 1.26 mmol) was added as a solid, followed by addition of 25% aq. NH_3_ (300 mL). The solution was heated to 60 °C and rapidly stirred for 2 h. After cooling to 23 °C, the solution was diluted with water and extracted with CH_2_Cl_2_. The organic phase was dried with sodium sulfate and filtered. The solvent was evaporated under reduced pressure, and the crude product was purified by silica gel column chromatography (10% EtOAc/hexane, purple fraction collected) and recrystallization from CH_2_Cl_2_/hexane (1:2 ratio) afforded pure **1-H**_**2**_ (54 mg, 33% yield) as a dark brown solid.
(e) Another attempt was performed by dissolving **CF**_**3**_**-TP** in regular methanol, into which 760 mg of ammonium carbamate was added instead of the 7 N NH_3_/methanol solution. **1-H**_**2**_ was collected as a dark brown solid (50 mg, 26% yield). Utilization of larger amounts of ammonium carbamate led to decreased yields.

^1^H NMR (400 MHz, CDCl_3_) δ (ppm): 9.28 ppm (2H, d, *J* = 5.1 Hz), 9.60 (2H, m), 9.71 (4H, m), −2.51 (2H, s). ^19^F NMR (377 MHz, CDCl_3_) δ (ppm): −36.43 (3F, s), and −38.18 (6F, s). Only the pyrrole-H resonance at 9.28 ppm appears as a classical doublet since the three others experience long-range ^19^F–H coupling with the meso-CF_3_ groups. HRMS (APCI, negative mode) for C_22_F_9_H_10_N_5_: 515.0802 (observed), 515.0792 (calculated). UV/vis (CH_2_Cl_2_) λ_max_/nm (ε, M^–1^ cm^–1^): 390 (49,206), 504 (3,175), 537 (10,318), 580 (1,984), 630 (11,111).

### Synthesis of [10,15,20-Tris(trifluoromethyl)-5-azaporphyrinato]zinc(II) [**1-Zn(H**_**2**_**O**)]

Zn(OAc)_2_·2H_2_O (458 mg, 0.25 mmol) dissolved in methanol (20 mL) was added to a solution of **1-H**_**2**_ (53 mg, 0.1 mmol) in CHCl_3_ (20 mL), and the mixture was heated to reflux and stirred for 2 h. After the mixture was cooled to room temperature and the solvent was evaporated under reduced pressure, the crude material was purified by silica gel flash chromatography (20% MeOH/EtOAc) to afford **1-Zn** as a purple solid (37 mg, 0.064 mmol, 62% yield). X-ray quality crystals were prepared by slow evaporation from a concentrated heptane/THF solution, layered by benzene. Diamagnetic ^1^H NMR (400 MHz, DMSO-*d*_6_) δ (ppm): 9.45 (2H, d, *J* = 4.9 Hz), 9.71 (2H, m), 9.78 (2H, s), 9.85 (2H, s). ^19^F NMR (377 MHz, DMSO-*d*_6_) δ (ppm): −36.44 (3F, s), −38.18 (6F, s). Only the pyrrole-H shift resonance at 9.45 ppm appears as a classical doublet since the three others experience long-range ^19^F–H coupling with the meso-CF_3_ groups. since the three others experience long-range ^19^F–H coupling with the meso-CF_3_ groups. HRMS (APCI, negative mode) for C_22_F_9_H_8_N_5_Zn: 576.9960 (observed), 576. 9927 (calculated). UV/vis (in THF): λ_max_/nm (ε, M^–1^ cm^–1^): 405 (106,000), 547 (9,000), 583 (32,000).

### Synthesis of [10,15,20-Tris(trifluoromethyl)-5-azaporphyrinato]cobalt(II) (**1-Co**)

A methanol solution (0.3 mL) of Co(OAc)_2_·4H_2_O (38 mg, 0.15 mmol) was added to a solution of **1-H**_**2**_ (7.8 mg, 0.015 mmol) in CHCl_3_ (2.2 mL), and the mixture was stirred and refluxed for 16 h. After being cooled to 23 °C and the solvent evaporation, the crude material was purified by silica column gel column chromatography (100% CHCl_3_, red fractions collected) to afford **1-Co** as a dark solid (8.2 mg, 0.0143 mmol, 95% yield). The complex was recrystallized from a concentrated CH_2_Cl_2_/hexane (1:3) solution. X-ray quality crystals were prepared by slow evaporation of a concentrated CH_2_Cl_2_/benzene (1:3) solution. Paramagnetic ^1^H NMR (400 MHz, CDCl_3_) δ (ppm): 13.83 (2H, br s), 15.39 (2H, br s), 17.16 (4H, br. s). ^19^F NMR (377 MHz, CDCl_3_) δ (ppm): −29.60 (6F, s), −68.82 (3F, s). HRMS (APCI, positive mode) for C_22_F_9_H_8_N_5_Co: 573.0042 (observed), 573.0046 (calculated). UV/vis (in DCM): λ_max_/nm (ε, M^–1^ cm^–1^): 388 (15,668), 571 (4,733).

### Synthesis of [10,15,20-Tris(trifluoromethyl)-5-azaporphyrinato]copper(II) (**1-Cu**)

An ethanol solution (20 mL) of Cu(OAc)_2_·H_2_O (1.15 g, 5.7 mmol) was added to a solution of **1-H**_**2**_ (120 mg, 0.23 mmol) in CH_2_Cl_2_ (20 mL), and the mixture was stirred and heated to reflux for 18 h. After cooling to 20 °C and the solvent evaporation, the crude material was purified by silica gel column chromatography (100% CHCl_3_, red fractions collected) to afford **1-Cu** as a dark purple solid (80 mg, 0.14 mmol, 61% yield). The complex was recrystallized from a CH_2_Cl_2_/hexane (1:3) solution. X-ray quality crystals were prepared by slow evaporation of a concentrated CH_2_Cl_2_/benzene (1:3) mixture. Paramagnetic NMR showed no peaks. HRMS (TOF-ESI) for C_22_F_9_H_8_N_5_Cu: 577.0011 (observed), 577.001 (calculated). UV/vis (in CH_2_Cl_2_): λ_max_/nm (ε, M^–1^ cm^–1^): 391 (950,455), 539 (7,801), 579 (31,915).

### Synthesis of [10,15,20-Tris(trifluoromethyl)-5-azaporphyrinato]nickel(II), (**1-Ni**)

A methanol solution (20 mL) of Ni(OAc)_2_·6H_2_O (1.02 g, 5.7 mmol) was added to a solution of **1-H**_**2**_ (120 mg, 0.23 mmol) in CHCl_3_ (20 mL), and the mixture was stirred and refluxed for 24 h. After cooling to 20 °C and the solvent evaporation under reduced pressure, the crude material was purified by silica gel column chromatography (100% CHCl_3_, red fractions collected) to afford **1-Ni** as a dark solid (25 mg, 0.044 mmol, 19% yield). The complex was recrystallized from a CH_2_Cl_2_/hexane (1:3) solution. X-ray quality crystals were prepared by slow evaporation of a concentrated CH_2_Cl_2_/benzene (1:3) solution. Diamagnetic ^1^H NMR (400 MHz, CDCl_3_) δ (ppm): 9.14 (2H, d, *J* = 5.2 Hz), 9.44 (4H, m), 9.50 (2H, m). ^19^F NMR (377 MHz, CDCl_3_) δ (ppm): −40.26 (6F, s), −40.31 (3F, s). Only the pyrrole-H resonance at 9.14 ppm appears as a classical doublet since the three others experience long-range ^19^F–H coupling with the meso-CF_3_ groups. HRMS (APCI, positive mode) for C_22_F_9_H_8_N_5_Ni: 571.9992 (observed), 572.0067 (calculated). UV/vis (in DCM): λ_max_/nm (ε M^–1^ cm^–1^): 391 (60,952), 535 (6,667), 580 (24,190).

### Synthesis of [10,15,20-Tris(trifluoromethyl)-5-azaporphyrinato]iron(III) Chloride (**1-FeCl**)

A methanol solution (20 mL) of FeCl_2_·4H_2_O (650 mg, 3.2 mmol) was added to a solution of **1-H**_**2**_ (65 mg, 0.13 mmol) in CHCl_3_ (20 mL), and the mixture was stirred and heated to reflux for 24 h. After cooling to room temperature and removing the solvent, the crude material was purified by silica gel column chromatography (20% MeOH/CHCl_3_, red fractions collected). The resulting solid was dissolved in CH_2_Cl_2_, washed 3 times with 10% aq. HCl and once with water, and dried over sodium sulfate, and the solvent was evaporated. The compound was crystallized from a CH_2_Cl_2_/hexane (1:1) solution to afford **1-FeCl** as a dark solid (25 mg, 0.016 mmol, 13% yield). X-ray quality crystals were grown by slow evaporation of a concentrated CH_2_Cl_2_/benzene (1:3) solution. Paramagnetic ^1^H NMR (400 MHz, CDCl_3_) δ (ppm): 84.68 (2H, br s), 86.68 (2H, br s), 90.23 (2H, br s), 92.52 (2H, br s). HRMS (APCI, negative mode) for C_22_F_9_H_8_N_5_FeCl: 603.9587 (observed), 603.9673 (calculated). UV/vis (in DCM): λ_max_ (ε M^–1^ cm^–1^): 343 (44682), 390 (34545), 488 (8909), 558 (6909), 643 (5864).

### Synthesis of [10,15,20-Tris(trifluoromethyl)-5-azaporphyrinato]iron(III) μ-Oxo Dimer (**1-Fe**_**2**_**O**)

A methanol solution (20 mL) of FeCl_2_·4H_2_O (1.93 g, 0.97 mmol) was added to a solution of **1-H**_**2**_ (200 mg, 0.39 mmol) in CHCl_3_ (20 mL), and the mixture was stirred and refluxed for 24 h. After the mixture was cooled to room temperature and the solvent removed, the crude material was purified by silica gel column chromatography (20% MeOH/CHCl_3_, red fractions collected). The resulting solid was dissolved in CH_2_Cl_2_, washed three times with aq. NaOH and once with water, and dried over sodium sulfate, and the solvent was evaporated. The compound was crystallized from a CH_2_Cl_2_/hexane (1:1) solution to afford **1-Fe**_**2**_**O** as a dark solid (25 mg, 0.02 mmol, 5% yield). X-ray quality crystals were grown by slow evaporation of a concentrated CH_2_Cl_2_/benzene (1:3) solution. Paramagnetic ^1^H NMR (400 MHz, CDCl_3_) δ (ppm): 14.50 (4H, br s), 15.34 (8H, br s), 15.67 (4H, br. s). ^19^F NMR (377 MHz, CDCl_3_) δ (ppm): −32.53 (12F, s), −33.49 (6F, s). HRMS (APCI, negative mode) for [(C_22_F_9_H_8_N_5_Fe)_2_O]: 1154.1063 (observed), 1153.9919 (calculated). UV/vis (in DCM): λ_max_ (ε M^–1^ cm^–1^): 340 (31846), 412 (26974), 593 (12410).

### Synthesis of 5,10,15-Tris(trifluoromethyl)porphyrin (**2-H**_**2**_) and 5,10,15,20-Tetrakis(trifluoromethyl)porphyrin (**3-H**_**2**_)

Synthesis was performed according to a published procedure^42^ while noting that a mistake therein. 5-trifluoromethyldipyrromethane that is listed as being used in the last step of the synthesis leads to 5,10,15,20-tetrakis(trifluoromethyl)porphyrin (**3-H**_**2**_). By using unsubstituted dipyrromethane, prepared by a previously reported procedure,^9^ the desired product was obtained after purification on a silica column with 10% ethyl acetate/hexane eluent.^42,43^**2-H**_**2**_ and **3-H**_**2**_ were synthesized in 9 and 5.5% yield, respectively. The ^1^H and ^19^F NMR data were identical to those reported in the published procedures.^42,43^

## Supporting Information Available

Methods, experimental details, crystal data, adsorption on both carbon supports, and comparison of ORR activity of each porphyrin on Vulcan and BP2000 (PDF)
