# Supplementary material for: Surprising Route to a Monoazaporphyrin and Full Characterization of Its Complexes with Five Different 3d Metals
Source: Inorg Chem. 2024 Apr 17;63(17):7828–37. doi: 10.1021/acs.inorgchem.4c00436 (PMC11061829; doi:10.1021/acs.inorgchem.4c00436)
Supplement: Supplementary file 1 — ic4c00436_si_001.pdf [file ic4c00436_si_001.pdf]

The Surprising Route to a Monoazaporphyrin and Full Characterization of its Complexes with Five Different 3d Metals

Arik Raslin,[a] Zachary P. Sercel,[b] Natalia Fridman,[a] Irena Saltsman,[a] and Zeev Gross*[a]

[a] Schulich Faculty of Chemistry, Technion–Israel Institute of Technology, Haifa 32000 Israel

[b] Division of Chemistry and Chemical Engineering, California Institute of Technology, Pasadena, California 91125, USA

Corresponding Author. Email: chr10zg@technion.ac.il

Table of Contents	Page
**Experimental details**	S2
**Table S1:** Crystallographic information for **1-Zn**, **1-FeCl**, **1-Fe_2O** and **1-Cu**.	S4
**Table S2:** Crystallographic information for **1-Ni** and **1-Co**.	S5
**Table S3:** Metal-nitrogen and metal-ligand (when relevant) bond lengths in the various complexes.	S6
**Table S4:** Complexes with disorder and their characteristics.	S6
**Figure S1:** HRMS of **1-H_2**, APCI negative mode.	S7
**Figure S2:** HRMS of **1-Co**, APCI positive mode.	S7
**Figure S3:** HRMS of **1-Zn**, APCI negative mode.	S8
**Figure S4:** HRMS of **1-Cu**, TOF ESI mode.	S8
**Figure S5:** HRMS of **1-Ni**, APCI negative mode.	S9
**Figure S6:** HRMS of **1-FeCl**, APCI negative mode.	S9
**Figure S7:** HRMS of **1-Fe_2O**, APCI negative mode.	S10
**Figure S8**. ^{1}H NMR spectrum (400 MHz, $CDCl_3$) of **1-H_2**	S10
**Figure S9**. ^{19}F NMR spectrum (377 MHz, $CDCl_3$) of **1-H_2**.	S11
**Figure S10**. ^{1}H NMR spectrum (400 MHz, $CDCl_3$) of **1-Co**.	S11
**Figure S11**. ^{19}F NMR spectrum (377 MHz, $CDCl_3$) of **1-Co**.	S12
**Figure S12**. ^{1}H NMR spectrum (400 MHz, $CDCl_3$) of **1-Ni**.	S12
**Figure S13**. ^{19}F NMR spectrum (377 MHz, $CDCl_3$) of **1-Ni**.	S13
**Figure S14**. ^{1}H NMR spectrum (400 MHz, $CDCl_3$) of **1-FeCl**.	S13
**Figure S15**. ^{1}H NMR spectrum (400 MHz, $CDCl_3$) of **1-Fe_2O**.	S14
**Figure S16**. ^{19}F NMR spectrum (377 MHz, $CDCl_3$) of **1-Fe_2O**.	S14
**Figure S17**. ^{1}H NMR spectrum (400 MHz, DMSO-d_6) of **1-Zn(OH_2).**	S15
**Figure S18**. ^{19}F NMR spectrum (377 MHz, DMSO-d_6) of **1-Zn(OH_2).**	S15
**Figure S19:** Electronic spectra of 0.8 mg adsorption of **1-Co** (red), **2-Co** (brown) and **3-Co** (yellow) on 10 mg a) Vulcan and b) BP2000 from 1 mL isopropanol.	S16
**References**	S16

Experimental details

Chemicals and Instrumentation

All reagents were purchased from commercial sources and used as received. BP2000, Vulcan XC- 72R and 20% Pt/ Vulcan were purchased from the FuelCellStore company. The stationary homogeneous and heterogeneous experiments were performed on a PALMSENS EmStat3+ potentiostat. Electronic spectra were recorded on a Cary 8454 spectrophotometer from Agilent. The fluorescence emission data was recorded on Fluorolog-3 spectrofluorometer equipped with a Xenon-arc lamp. Single-crystal materials were immersed in Paratone-N oil and mounted on a Bruker APEX II diffractometer at 100 K. ^{1}H and ^{19}F NMR spectra were recorded on Bruker Avance III 400 spectrometer (400 MHz for ^{1}H and 377 MHz for ^{19}F) and the chemical shifts are reported in ppm relative to residual hydrogen atoms of $CDCl_3$ solvent, unless mentioned otherwise.

Crystallographic details

The single-crystal of dark violet material were immersed in Paratone–N oil and mounted on a Rigaku Oxford Diffraction – XtaLAB Synergy-S at 100.15 K for **1-Zn(H_2O)**, **1-FeCl**, **1-Fe_2O**, and **1-Ni**, while for **1-Co** and **1-Cu** at 140.15 K. Data collection was performed using monochromated Mo Kα radiation, at λ = 0.71073 Å, using φ and ω scans to cover the Ewald sphere. Accurate cell parameters were obtained with the amount of indicated reflections. Using Olex2[1], the structure was solved with the olex2.solve[2] structure solution program using Charge Flipping and refined with the ShelXL[3] refinement package using Least Squares minimization. All non-hydrogen atoms were refined with anisotropic displacement parameters. The hydrogen atoms were refined isotropically on calculated positions using a riding model with their U_{iso} values constrained to 1.5 times the U_{eq} of their pivot atoms for terminal sp^3 carbon atoms and 1.2 times for all other carbon atoms. Software used for molecular graphics: Mercury 2022.3.0.[4] We used the SIMU, DELU restrictions for partly disordered crystals: 31R, 43R, 45R, 29R.

Cyclic Voltammograms were performed in acetonitrile or dichloromethane solutions of 0.5 mM complex and 0.1 M tetrabutylammonium hexafluorophosphate ($TBAPF_6$), equipped with a glassy carbon working electrode, and Ag/$AgNO_3$ and Pt wire as reference and counter electrodes, respectively. The solutions were purged with nitrogen prior to all experiments.

ORR Catalysis by Catalyst-Modified Electrodes: The RRDE measurements were performed by a four-electrode set up, with a 4 mm glassy carbon disc and a 7 mm Pt ring as working electrodes, Ag/AgCl as reference electrode and a Pt counter electrode. 10 µL of ink were drop casted on the glassy carbon surface and dried. All ORR experiments were performed in 0.1 M KOH solution. Before each measurement, the solution was purged with nitrogen for 30 minutes

and wetting of the working electrode was performed. Then, the solution was purged with oxygen for 30 minutes. The ring potential was kept at 0.96 V vs. RHE. The measurements were performed at 1000 rpm, 20 mV/s. The collection efficiency, N, of the electrode was calculated by RRDE experiment of a 2 mM $K_3[Fe(CN)_6]$ + 0.1 M KNO_3 aqueous solution under nitrogen atmosphere. It was found to be 0.448. The percentage of peroxide ions was calculated by equation S1:

S1) $$\%H_2O_2 = \frac{I_R}{I_D \times N} \times 100\%$$

where I_R is ring current, I_D is disc current and N is the collection efficiency of the RRDE.[3,4]

Adsorption of the complexes on Vulcan/BP2000, ink preparation and its adsorption on the working electrode: 0.8 mg of metallocorrole was dissolved in 1 mL of isopropanol (IPA) by five-minute sonication. 10 mg Vulcan or BP2000 were added, and the solution was sonicated for 15 minutes and left for 24 hours stirring. After that, the catalyst was centrifuged for thirty minutes, the solution was separated, and the carbon support was dried at 45 °C in an oven overnight. Then, 1 mL of IPA was added, and the mixture was centrifuged again. The solution was removed and combined with the previous day's solution. The catalyst was dried overnight again at the same temperature. The ink consisted of 1 mg catalyst, 0.2 mL IPA, 0.8 mL doubly ionized (DI) water and 10 µL Nafion. The ink was sonicated for 30 minutes. 10 µL of the ink were drop casted on the working electrode, dried under air for 30 minutes and then at 45 °C in the oven for 30 minutes.

Table S1: Crystallographic information for **1-Zn**, **1-FeCl**, **1-Fe$_2$O** and **1-Cu**.

**Crystal data**	**Gross 31R 1-Zn**	**Gross 43R 1-FeCl**	**Gross 45R 1-Fe$_2$O**	**Gross 29R 1-Cu**
CCDC number	2296390	2296391	2296392	2296404
Empirical formula	$C_{26}H_{18}F_9N_5O_2Zn$	$C_{25}H_{11}ClF_9FeN_5$	$C_{44}H_{16}F_{18}Fe_2N_{10}O$	$C_{22}H_8CuF_9N_5$
Formula weight (gr/mol)	668.82	643.69	1154.37	576.87
Temperature (K)	100.15	100.15	100.15	140.15
Wavelength (Å)	0.71073	0.71073	0.71073	0.71073
Crystal system	triclinic	triclinic	Triclinic	triclinic
Space group	P-1	P-1	P-1	P-1
a (Å)	8.1590(2)	9.0716(2)	14.1094(3)	12.1543(4)
b (Å)	12.5479(5)	11.3114(3)	16.1128(4)	13.4976(4)
c (Å)	12.9352(4)	11.6301(3)	20.9251(4)	14.5675(5)
α (°)	77.752(3)	96.494(2)	69.839(2)	117.142(4)
β (°)	75.288(4)	91.031(3)	87.4571(17)	111.202(3)
γ (°)	75.799(5)	102.175(4)	66.528(2)	90.176(3)
Volume (Å^3)	1225.77(8)	1157.99(5)	4072.24(18)	1941.62(13)
Z	2	2	4	4
Calculated density (g/cm^3)	1.812	1.846	1.883	1.973
Absorption coefficient (mm^{-1})	1.109	0.868	0.850	1.236
F (000)	672.0	640.0	2288.0	1140.0
Crystal size (mm^3)	0.33×0.12×0.09	0.21×0.15×0.06	0.36×0.12×0.09	0.3×0.12×0.09
2Θ range (°)	5.096-60.078	4.804-59.812	4.75-59.84	4.524-60.166
Reflections collected	14892	18818	52467	17067
R_{int}	0.0534	0.0396	0.0559	0.0400
Data/restraints/ parameters	5561/429/399	5338/3/398	18281/1854/1513	8235/832/695
Goodness-of-fit on F^2	0.992	1.038	1.023	1.015
R_1, wR_2 [I> 2σ (I)]	0.0437, 0.1015	0.0318, 0.0714	0.0417, 0.0861	0.0412, 0.0865
R_1. wR_2 [all data]	0.0639, 0.1090	0.0453, 0.0754	0.0710, 0.0943	0.0671, 0.0960
Largest diff. peak/ hole/ e Å^{-3}	0.69/-0.45	0.41/-0.33	0.41/-0.49	0.64/-0.47
Diffractometer	Rigaku XtaLAB Synergy-S	Rigaku XtaLAB Synergy-S	Rigaku XtaLAB Synergy-S	Rigaku XtaLAB Synergy-S

Table S2: Crystallographic information for **1-Ni** and **1-Co**.

**Crystal data**	**Gross 34R<br>1-Ni**	**Gross 1R<br>1-Co**
CCDC number	2296394	2296389
Empirical formula	$C_{22}H_8F_9N_5Ni$	$C_{22}H_8CoF_9N_5$
Formula weight (gr/mol)	572.04	572.26
Temperature (K)	100.15	140.15
Wavelength (Å)	0.71073	0.71073
Crystal system	triclinic	triclinic
Space group	P-1	P-1
a (Å)	12.0615(3)	12.2205(2)
b (Å)	13.8662(3)	13.5302(2)
c (Å)	14.2366(3)	14.1720(3)
α (°)	116.973(2)	115.4873(18)
β (°)	110.352(3)	110.9276(18)
γ (°)	90.761(4)	90.5835(15)
Volume ($Å^3$)	1947.43(9)	1938.02(7)
Z	4	4
Calculated density (g/cm^3)	1.951	1.961
Absorption coefficient (mm^{-1})	1.107	0.997
F (000)	1136.0	1132.0
Crystal size (mm^3)	0.33×0.12×0.09	0.24×0.15×0.15
2Θ range (°)	4.522-59.13	4.47-59.468
Reflections collected	23392	28085
R_{int}	0.0339	0.0364
Data/restraints/ parameters	8784/772/667	8920/772/667
Goodness-of-fit on F^2	1.040	1.059
R_1, wR_2 [I> 2σ (I)]	0.0325, 0.0735	0.0335, 0.0785
R_1. wR_2 [all data]	0.0432, 0.0779	0.0421, 0.0826
Largest diff. peak/ hole/ e $Å^{-3}$	0.40/-0.35	0.44/-0.41
Diffractometer	Rigaku XtaLAB Synergy-S	Rigaku XtaLAB Synergy-S

Table S3: Metal-nitrogen and metal-ligand (when relevant) bond lengths in the various complexes.

	**1-Co**	**1-Cu**	**1-Ni**	**1-Zn**	**1-FeCl**	**1-Fe_2O**	
**M-N_1**	1.9319(16)	1.963(3)	1.9157(17)	2.061(2)	2.0500(15)	2.066(2)	2.056(2)
**M-N_2**	1.9293(15)	1.972(2)	1.9174(16)	2.052(2)	2.0528(16)	2.068(2)	2.057(2)
**M-N_3**	1.9273(16)	1.959(3)	1.9061(17)	2.077(2)	2.0382(15)	2.066(2)	2.048(2)
**M-N_4**	1.9253(15)	1.966(2)	1.9018(16)	2.062(2)	2.0431(15)	2.0662(19)	2.0515(19)
**M-Ligand**	-	-	-	2.0754(18)	2.2019(5)	1.7659(16)	1.7631(16)

Table S4: Complexes with disorder and their characteristics

	**Part 1**	**Part 2**
**Gross 31R**<br>**1-Zn**	C_{24}-C_{24A}<br>0.78/0.22	-
**Gross 43R**<br>**1-FeCl**	F_4-F_6<br>0.71	F_{4a}-F_{6a}<br>0.29
**Gross 45R**<br>**1-Fe_2O**	F_{10}-F_{12} 0.72<br>F_{10C}-F_{12C} 0.28<br>F_4-F_6 0.54<br>F_{4C}-F_{6C} 0.46<br>F_7-F_9 0.74<br>F_{7C}-F_{9C} 0.26<br>F_{7A}-F_{9A} 0.85<br>F_{7B}-F_{9B} 0.15<br>F_{10A}-F_{12A} 0.57<br>F_{10B}-F_{12B} 0.43	-
**Gross 29R**<br>**1-Cu**	F_{4A}-F_{6A} 0.53<br>F_{4C}-F_{6C} 0.47	-

Analysis Info

Analysis Name	D:\Data\Gross\Gr_3423n000001.d	Acquisition Date	03/08/2022 15:52:39
Method	APCI_pos_SolidProbe.m	Operator	Larisa Panz
Sample Name	N porphyrin	Instrument	maXis impact 282001.00128
Comment			

Acquisition Parameter

Source Type	APCI	Ion Polarity	Negative	Set Nebulizer	1.2 Bar
Focus	Active	Set Capillary	4000 V	Set Dry Heater	120 °C
Scan Begin	50 m/z	Set End Plate Offset	-500 V	Set Dry Gas	1.5 l/min
Scan End	2500 m/z	Set Charging Voltage	2000 V	Set Divert Valve	Source
		Set Corona	2000 nA	Set APCI Heater	250 °C

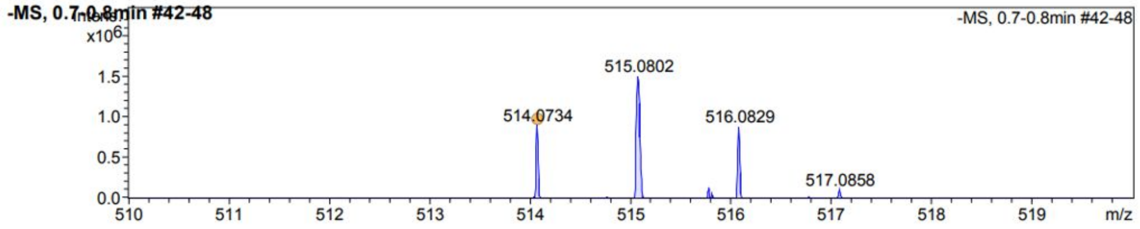

Figure S1: HRMS of **1-H$_2$**, APCI negative mode.

Analysis Info

Analysis Name	D:\Data\Gross\Gr_3499000001.d	Acquisition Date	22/08/2022 14:35:27
Method	APCI_pos_SolidProbe.m	Operator	Larisa Panz
Sample Name	CO-azaporphyrin	Instrument	maXis impact 282001.00128
Comment			

Acquisition Parameter

Source Type	APCI	Ion Polarity	Positive	Set Nebulizer	1.2 Bar
Focus	Active	Set Capillary	3000 V	Set Dry Heater	120 °C
Scan Begin	50 m/z	Set End Plate Offset	-500 V	Set Dry Gas	1.5 l/min
Scan End	2000 m/z	Set Charging Voltage	2000 V	Set Divert Valve	Source
		Set Corona	5000 nA	Set APCI Heater	300 °C

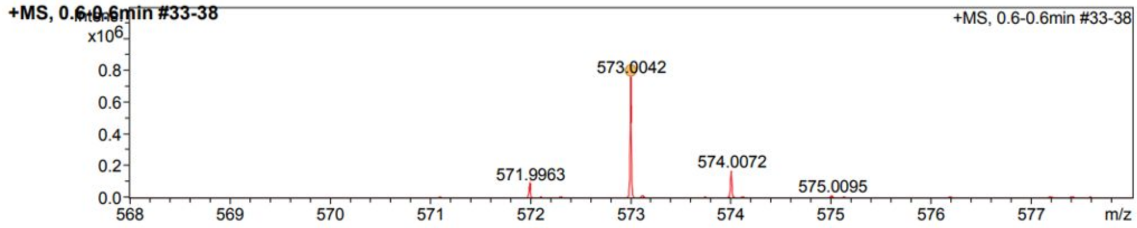

Figure S2: HRMS of **1-Co**, APCI positive mode.

Analysis Info

Analysis Name	D:\Data\Gross\Gr_3242 .d	Acquisition Date	06/07/2022 18:50:49
Method	APCI_pos_SolidProbe.m	Operator	Larisa Panz
Sample Name	ZPS I 58	Instrument	maXis impact 282001.00128
Comment			

Acquisition Parameter

Source Type	APCI	Ion Polarity	Negative	Set Nebulizer	1.2 Bar
Focus	Active	Set Capillary	4000 V	Set Dry Heater	120 °C
Scan Begin	50 m/z	Set End Plate Offset	-500 V	Set Dry Gas	1.5 l/min
Scan End	2000 m/z	Set Charging Voltage	2000 V	Set Divert Valve	Source
		Set Corona	2000 nA	Set APCI Heater	200 °C

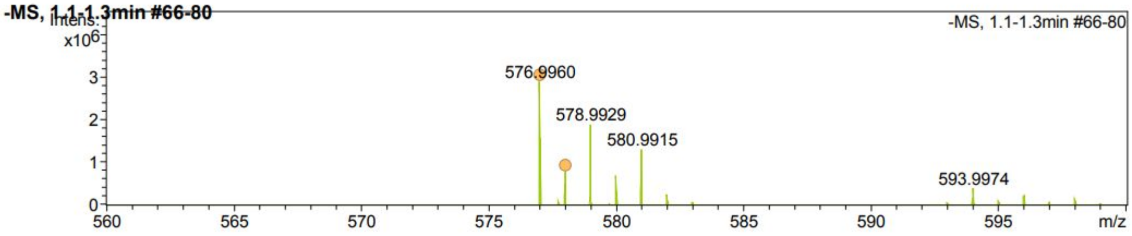

Figure S3: HRMS of **1-Zn(OH$_2$)**, APCI negative mode.

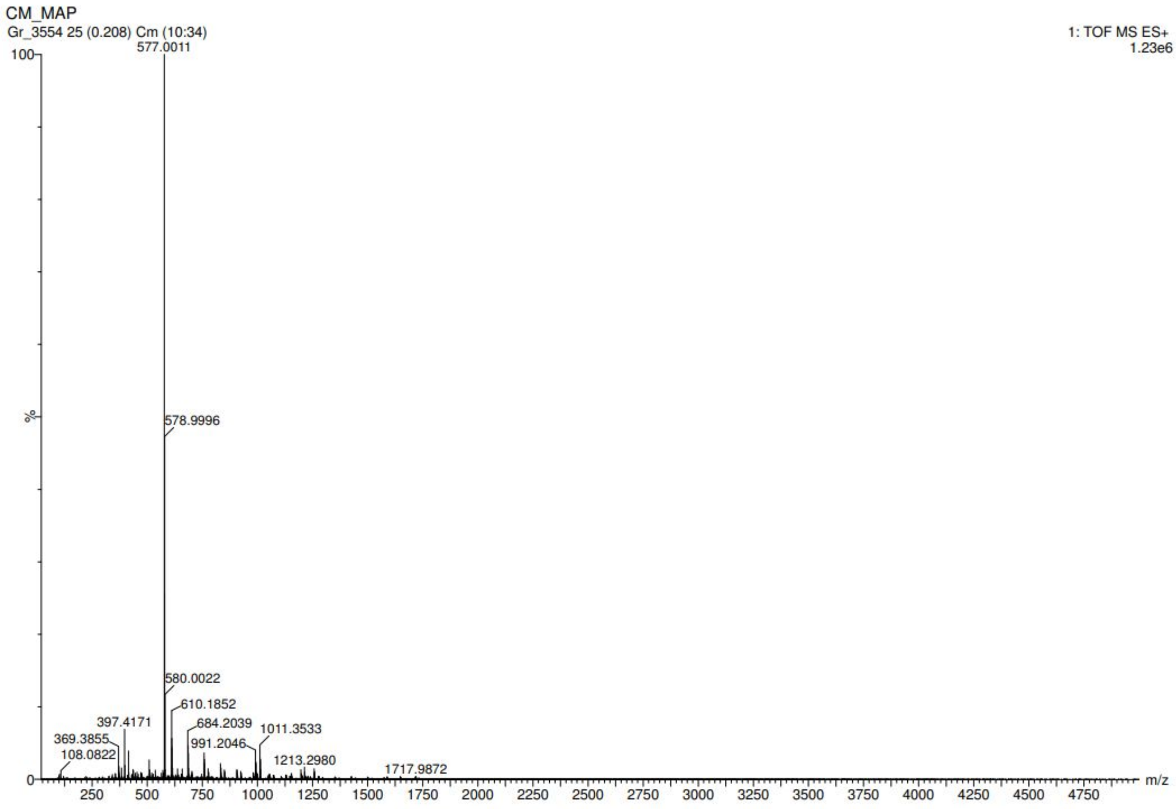

Figure S4: HRMS of **1-Cu**, TOF ESI mode.

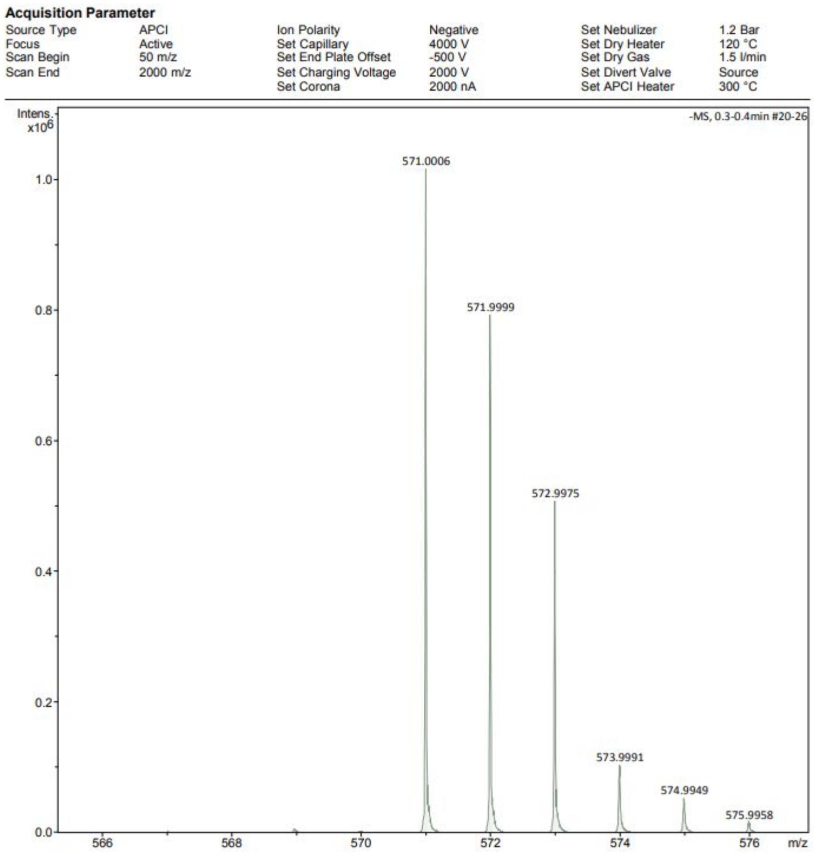

Figure S5: HRMS of **1-Ni**, APCI negative mode.

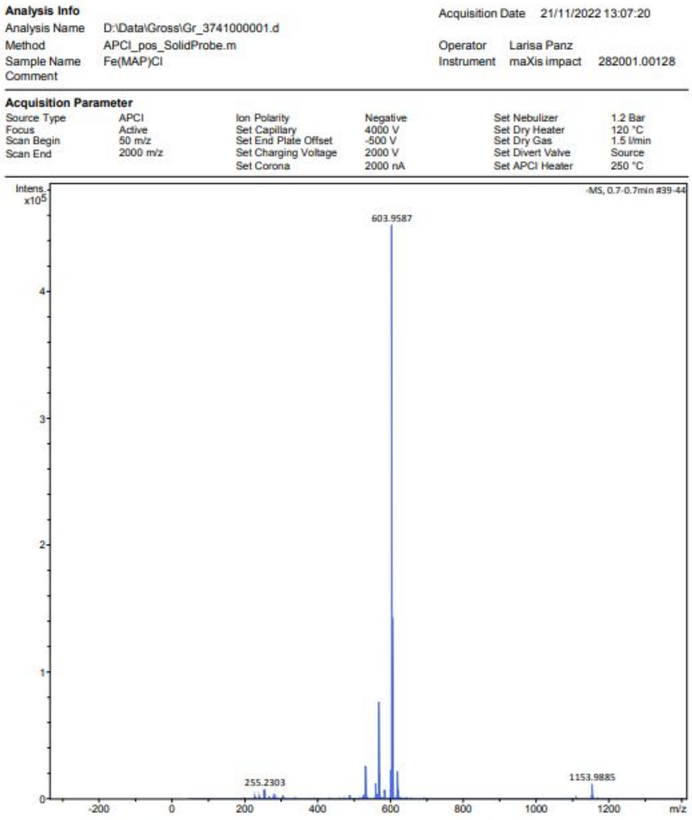

Figure S6: HRMS of **1-FeCl**, APCI negative mode.

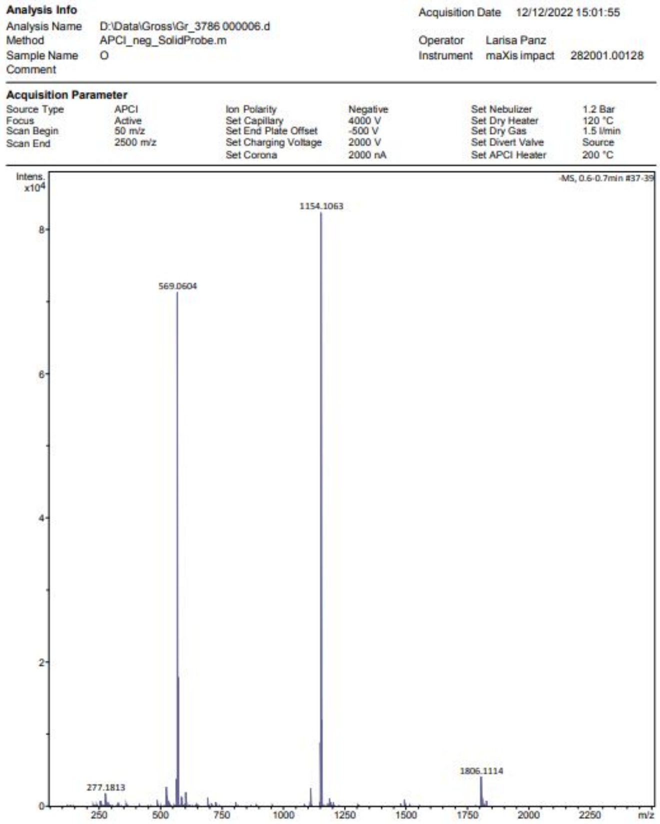

Figure S7: HRMS of **1-Fe₂O**, APCI negative mode.

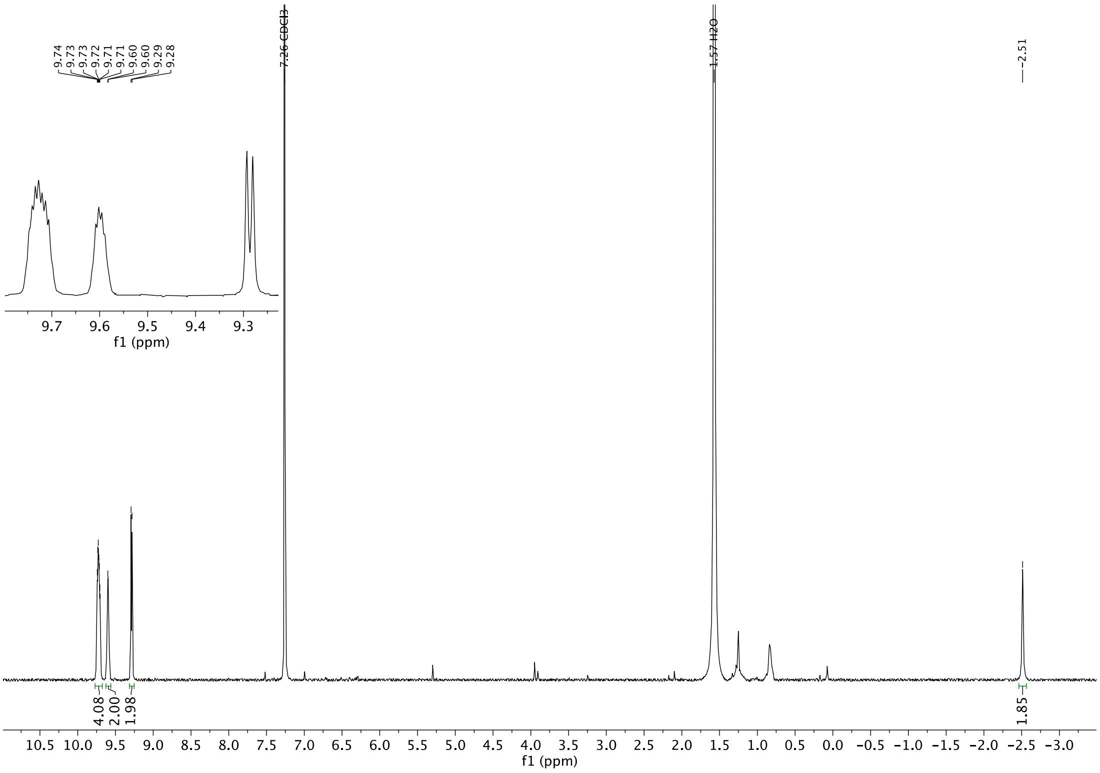

Figure S8. ^{1}H NMR spectrum (400 MHz, $CDCl_3$) of **1-H₂**.

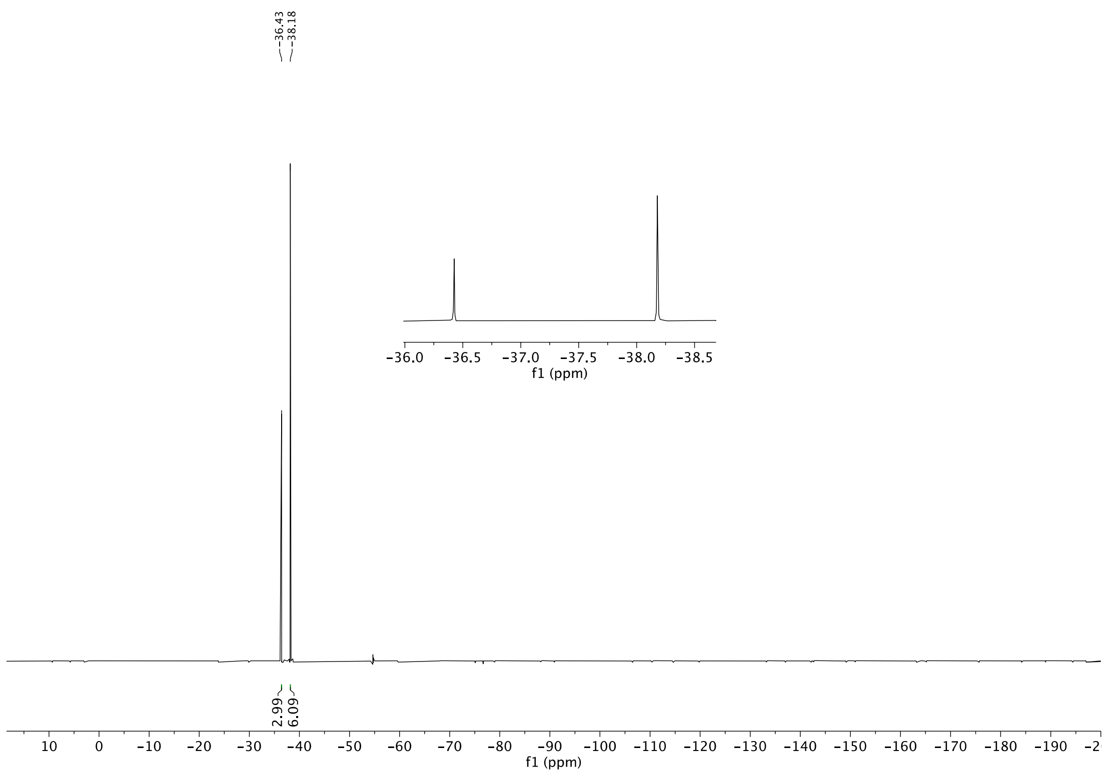

Figure S9. ^{19}F NMR spectrum (377 MHz, $CDCl_3$) of **1-H_2**.

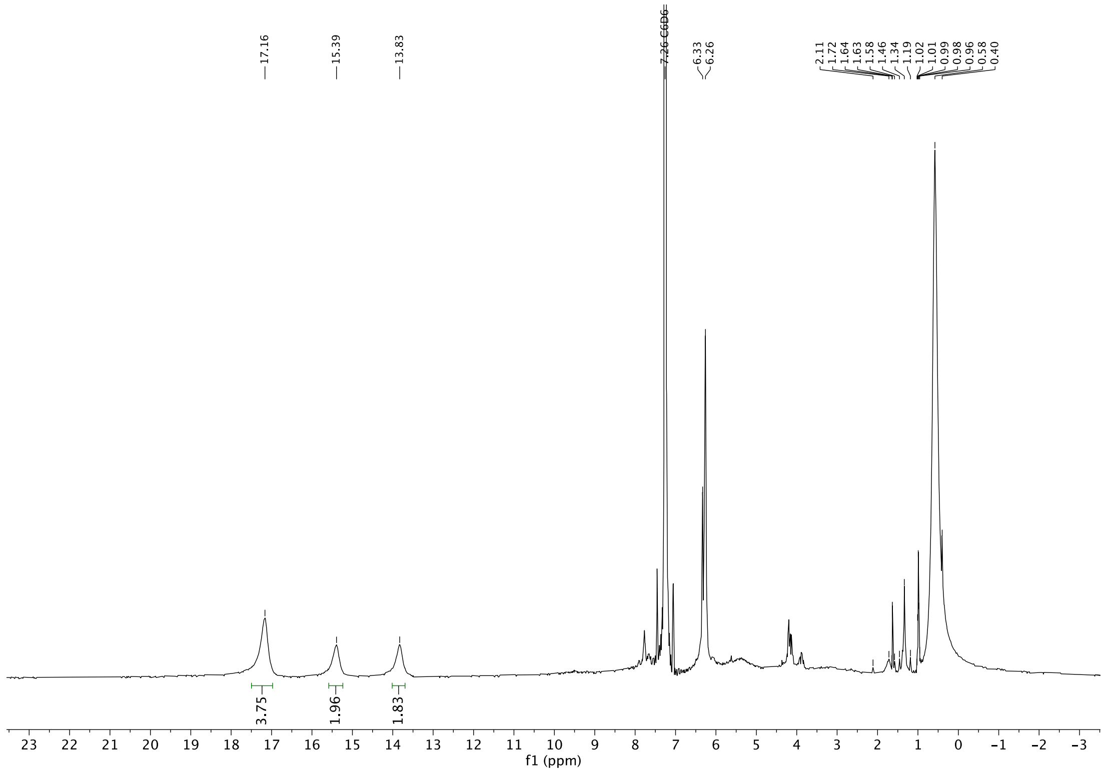

Figure S10. ^{1}H NMR spectrum (400 MHz, $CDCl_3$) of **1-Co**.

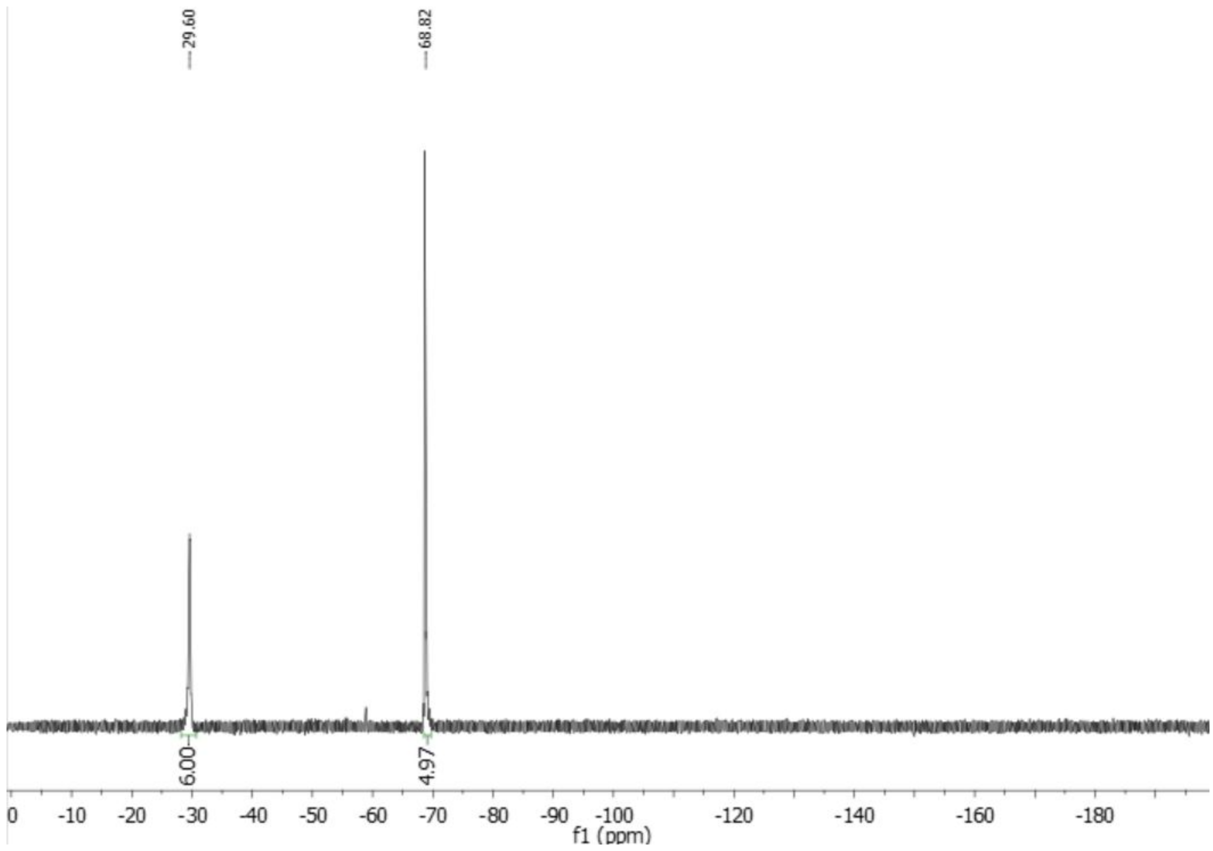

Figure S11. ^{19}F NMR spectrum (377 MHz, $CDCl_3$) of **1-Co**.

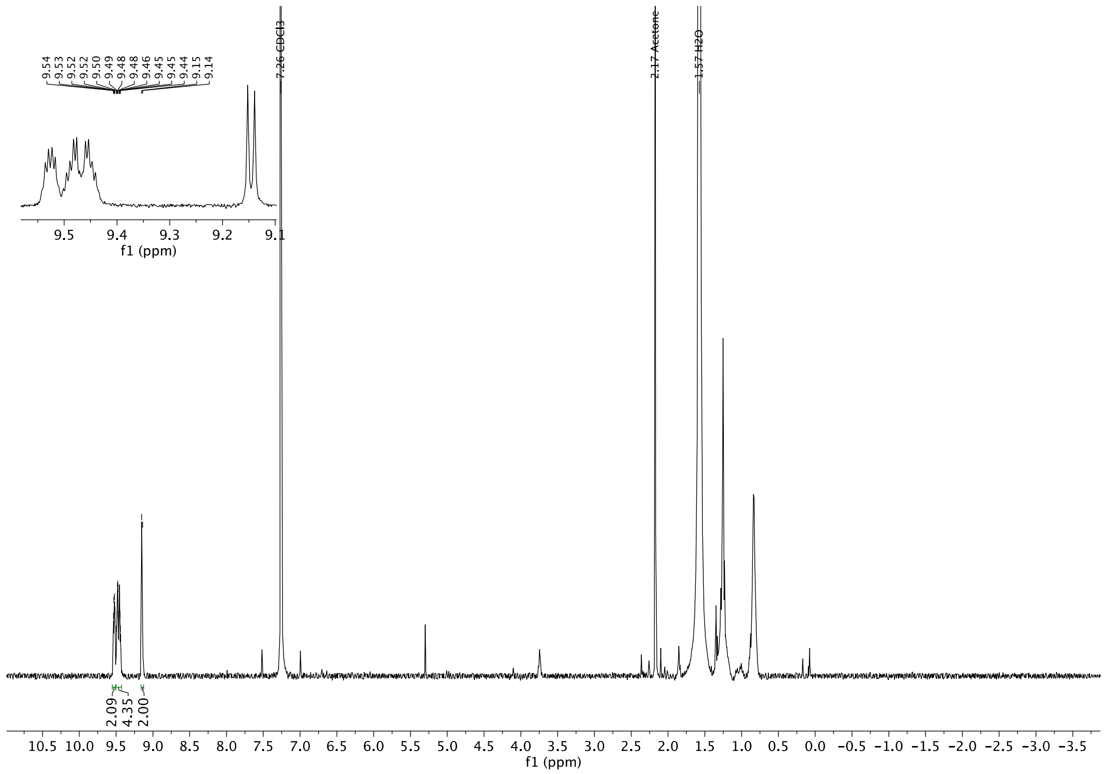

Figure S12. ^{1}H NMR spectrum (400 MHz, $CDCl_3$) of **1-Ni**.

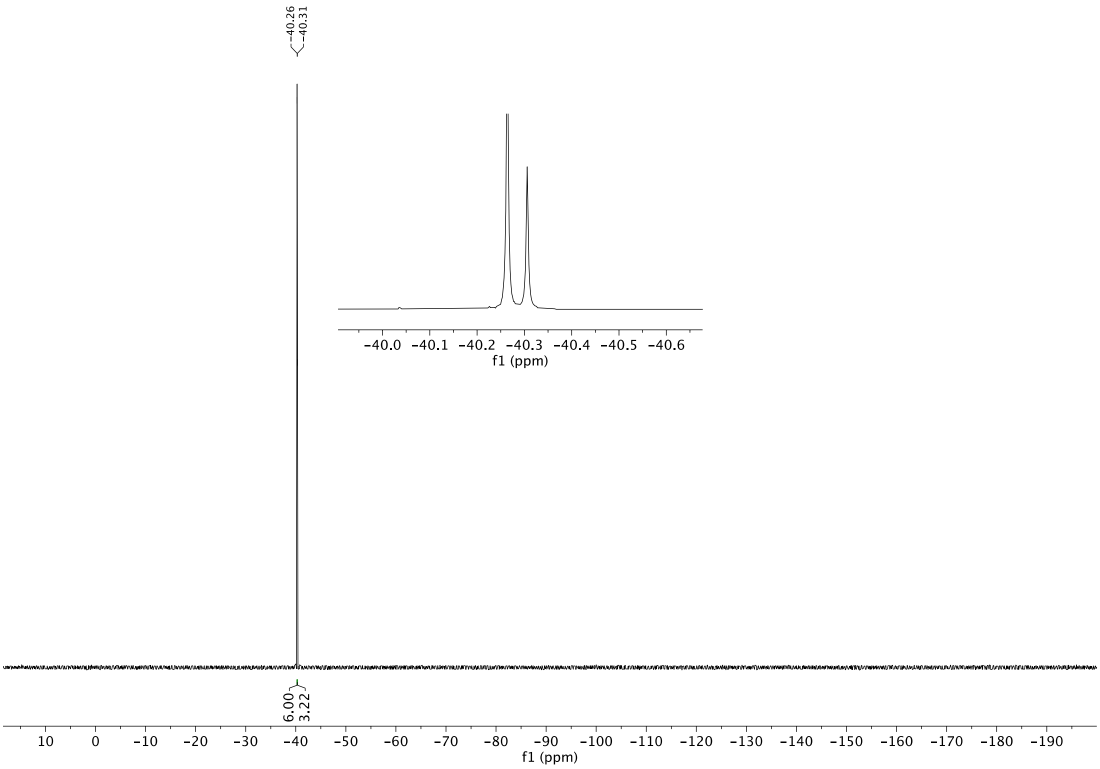

Figure S13. ^{19}F NMR spectrum (377 MHz, $CDCl_3$) of **1-Ni**.

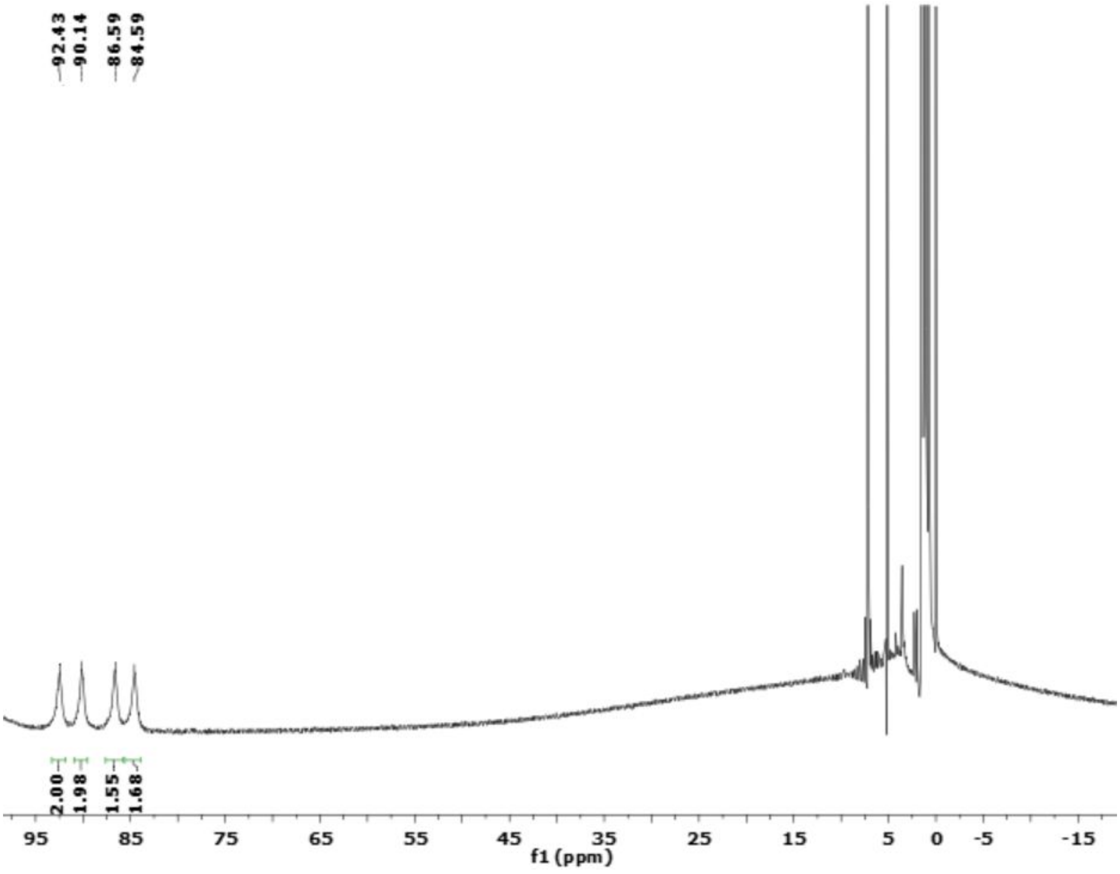

Figure S14. ^{1}H NMR spectrum (400 MHz, $CDCl_3$) of **1-FeCl**.

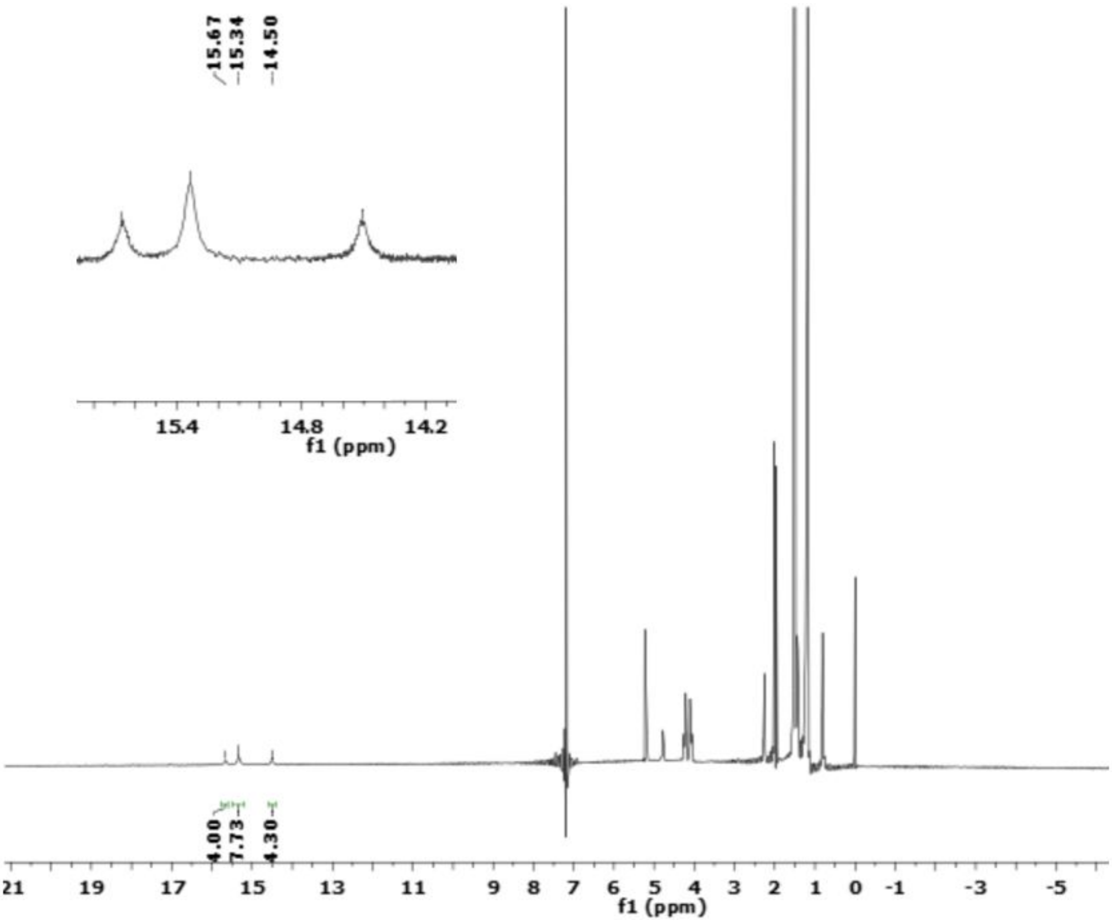

Figure S15. ^{1}H NMR spectrum (400 MHz, $CDCl_3$) of **1-Fe$_2$O**.

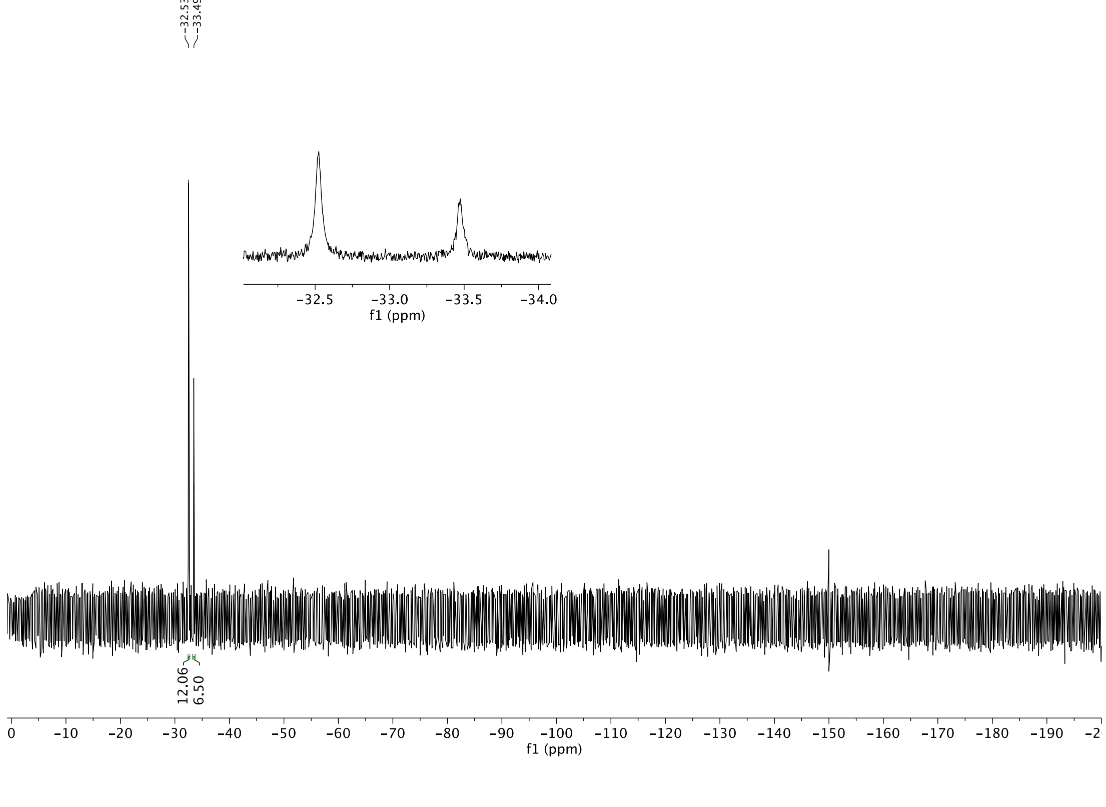

Figure S16. ^{19}F NMR spectrum (377 MHz, $CDCl_3$) of **1-Fe_2O**.

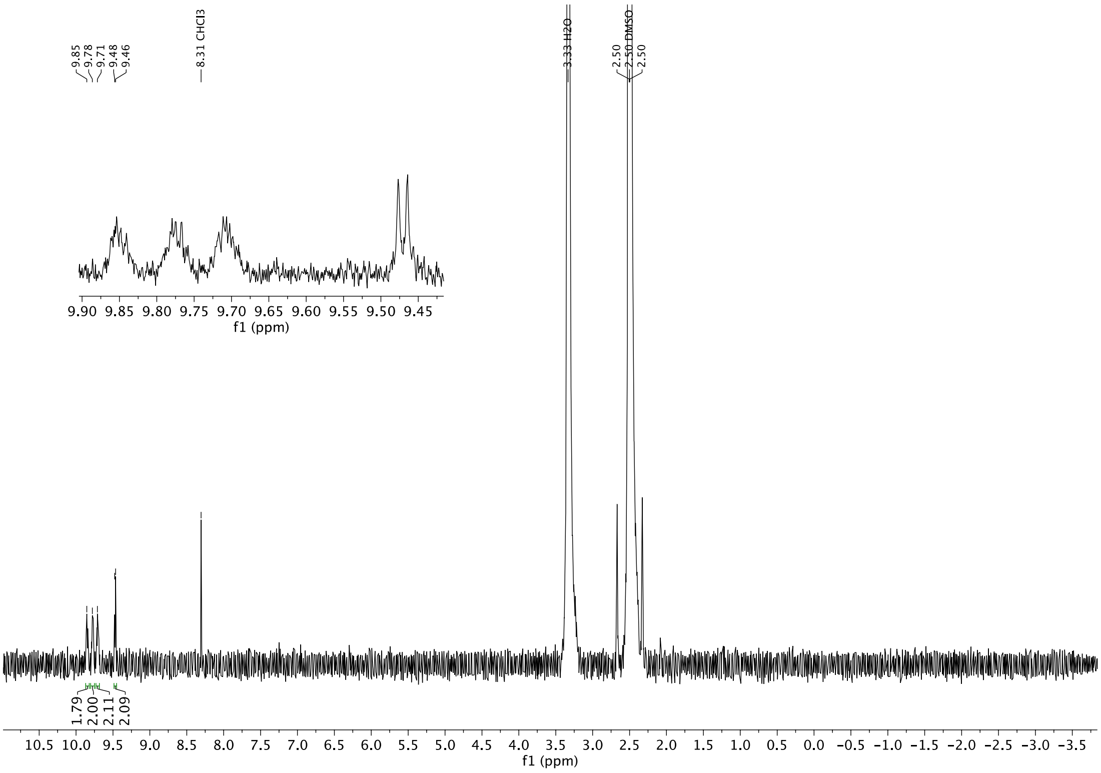

Figure S17. ^{1}H NMR spectrum (400 MHz, DMSO-d_6) of **1-Zn(OH_2)**.

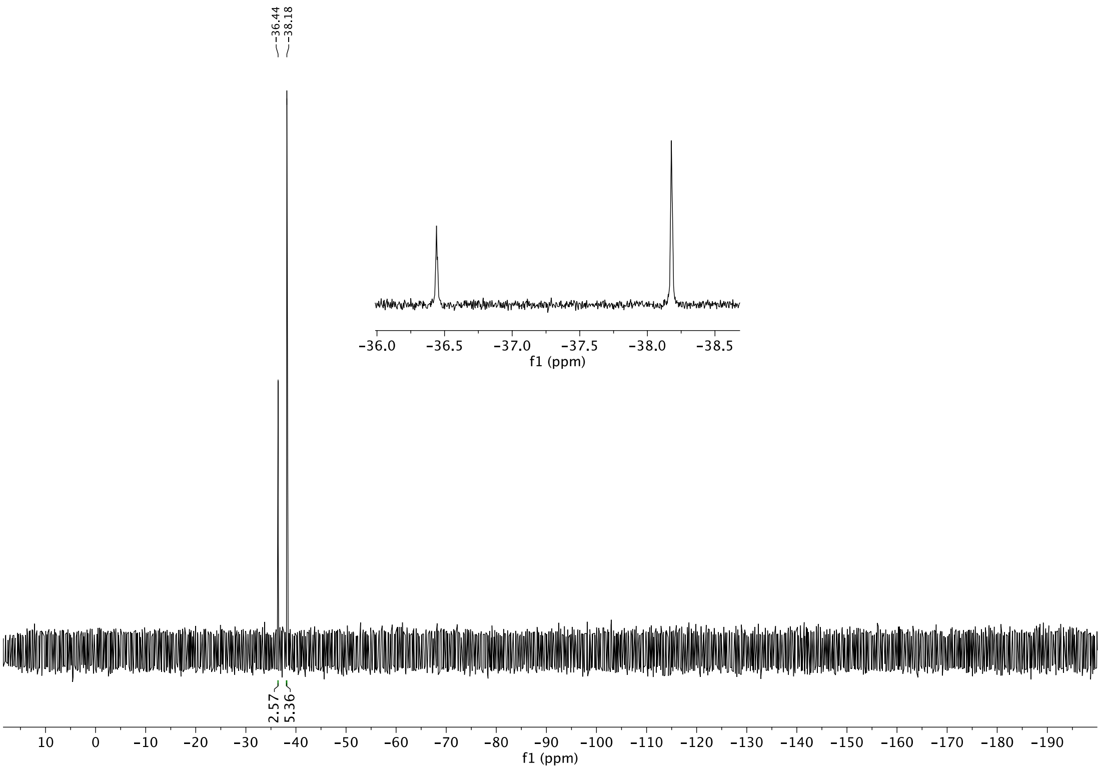

Figure S18. ^{19}F NMR spectrum (377 MHz, DMSO-d_6) of **1-Zn(OH_2)**.

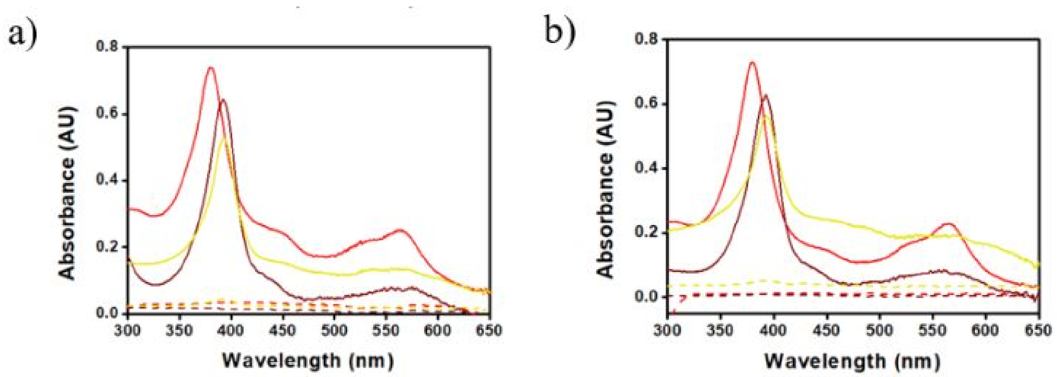

Figure S19: Electronic spectra of 0.8 mg adsorption of **1-Co** (red), **2-Co** (brown) and **3-Co** (yellow) on 10 mg a) Vulcan and b) BP2000 from 1 mL isopropanol.

References

(1) Dolomanov, O. V; Bourhis, L. J.; Gildea, R. J.; Howard, J. A. K.; Puschmann, H. OLEX2: A Complete Structure Solution, Refinement and Analysis Program. *J. Appl. Crystallogr.* **2009**, *42*, 339–341.

(2) Bourhis, L. J.; Dolomanov, O. V; Gildea, R. J.; Howard, J. A. K.; Puschmann, H. The Anatomy of a Comprehensive Constrained, Restrained Refinement Program for the Modern Computing Environment–Olex2 Dissected. *Acta Crystallogr. Sect. A Found. Adv.* **2015**, *71*, 59–75.

(3) Liberman, I.; Shimoni, R.; Ifraemov, R.; Rozenberg, I.; Singh, C.; Hod, I. Active-Site Modulation in an Fe-Porphyrin-Based Metal–Organic Framework through Ligand Axial Coordination: Accelerating Electrocatalysis and Charge-Transport Kinetics. *J. Am. Chem. Soc.* **2020**, *142*, 1933–1940.

(4) Honig, H. C.; Friedman, A.; Zion, N.; Elbaz, L. Enhancement of the Oxygen Reduction Reaction Electrocatalytic Activity of Metallo-Corroles Using Contracted Cobalt(Iii) CF3-Corrole Incorporated in a High Surface Area Carbon Support. *Chem. Commun.* **2020**, *56*, 8627–8630.
